# Temporal and spatiotemporal soliton molecules in ultrafast fibre lasers

**DOI:** 10.1515/nanoph-2024-0590

**Published:** 2025-02-05

**Authors:** Ding Mao, Zichuan Yuan, Ke Dai, Yue Chen, Huihui Ma, Qiang Ling, Jiancheng Zheng, Yusheng Zhang, Daru Chen, Yudong Cui, Zhipei Sun, Boris A. Malomed

**Affiliations:** Hangzhou Institute of Advanced Studies, Zhejiang Normal University, Hangzhou 311231, China; College of Information Engineering, Sanming University, Sanming 365004, China; Key Laboratory of Optical Information Detection and Display Technology of Zhejiang, Zhejiang Normal University, Jinhua 321004, China; State Key Laboratory of Extreme Photonics and Instrumentation, College of Optical Science and Engineering, Zhejiang University, Hangzhou 310027, China; ZJU-Hangzhou Global Scientific and Technological Innovation Center, Hangzhou 311200, Zhejiang, China; Department of Electronics and Nanoengineering and QTF Centre of Excellence, Aalto University, Espoo, Tietotie 3, FI-02150, Finland; Department of Physical Electronics, School of Electrical Engineering, Faculty of Engineering, Tel Aviv University, Tel Aviv 69978, Israel; Instituto de Alta Investigación, Universidad de Tarapacá, Casilla 7D, Arica, Chile

**Keywords:** soliton molecules, bound states, stability, fibre lasers, nonlinear dynamics, ultrafast nonlinear optics

## Abstract

Ultrafast fibre lasers, characterized by ultrashort pulse duration and broad spectral bandwidth, have drawn significant attention due to their vast potential across a wide range of applications, from fundamental scientific to industrial processing and beyond. As dissipative nonlinear systems, ultrafast fibre lasers not only generate single solitons, but also exhibit various forms of spatiotemporal soliton bunching. Analogous to molecules composed of multiple atoms in chemistry, soliton molecules (SMs) – alias bound states – in ultrafast fibre lasers are a key concept for gaining a deeper understanding of nonlinear interaction and hold a promise for advancing high-capacity fibre-optic communications. SMs are particularly notable for their high degree of controllability, including their internal temporal separation, and relative phase differences, thereby suggesting new possibilities for manipulating multi-pulse systems. In this review, we provide a comprehensive overview of recent advancements in the studies of SMs with the multidimensional parameter space in ultrafast fibre lasers. Owing to the flexibility afforded by mode-locking techniques and dispersion management, various types of SMs – with diverse values of the soliton number, relative phase, pulse separation, carrier frequencies, and even modal dispersion – have been experimentally demonstrated. We also discuss other basic nonlinear optical phenomena observed in fibre lasers, including the formation, spatiotemporal pulsations, and interaction dynamics of SMs. Furthermore, we explore the multidimensional control of SMs through approaches such as gain modulation, polarization control, dispersion management, and photomechanical effects, along with their applications to optical data encoding. Finally, we discuss challenges and future development of multidimensional technologies for the manipulation of SMs.

## 1 Introduction

Ultrashort pulse lasers, known for their extremely high electric-field amplitudes, energy densities, and ultrashort time scales, serve as the foundation for various basic laser applications, including optical communications, precision spectroscopy, biomedical imaging, material microprocessing, etc. [1], [2], [3], [4], [5]. These lasers enable measurements on ultrafast temporal scales that extend into the femtosecond and even attosecond ranges [6], [7], [8]. Passive mode-locked fibre lasers, which support optical solitons, offer the flexibility for reconfiguring ultrashort pulses [2], [9]. By carefully controlling cavity parameters, such as the dispersion, nonlinearity, gain, and loss, these lasers can generate a variety of soliton states, making them an ideal platform for studying the nonlinear dynamics of solitons, in addition to the diverse applications [9], [10].

Solitons, as localized waves in nonlinear systems, have drawn great interest across many disciplines, including fluid mechanics, plasma physics, optics, solid state, and quantum matter [11], [12], [13], [14], [15], [16], [17], [18], [19]. In nonlinear optical systems, such as fibre lasers, multiple solitons can interact and bind together to form soliton molecules (SMs) or bound states [20], [21], [22], [23], [24], [25], [26], characterized by a constant temporal separation and a fixed phase difference [27]. Multi-soliton and complex states include soliton compounds [20], bright and dark soliton modes [28], vector solitons [29], soliton rains [30], rogue waves [31], and coexisting dissipative solitons [32]. On the one hand, SMs exhibit various spatiotemporal dynamical scenarios, such as vibrations, fusion and fission, and others, similar to the behaviour of atomic molecules [33], [34]. Understanding the transient dynamics of the SMs helps to refine the theoretical framework for the work with solitons. On the other hand, SMs hold the potential for enhancing communication capacity and advancing all-optical data-storage technologies [35], [36], [37], [38].

**Figure 1:**
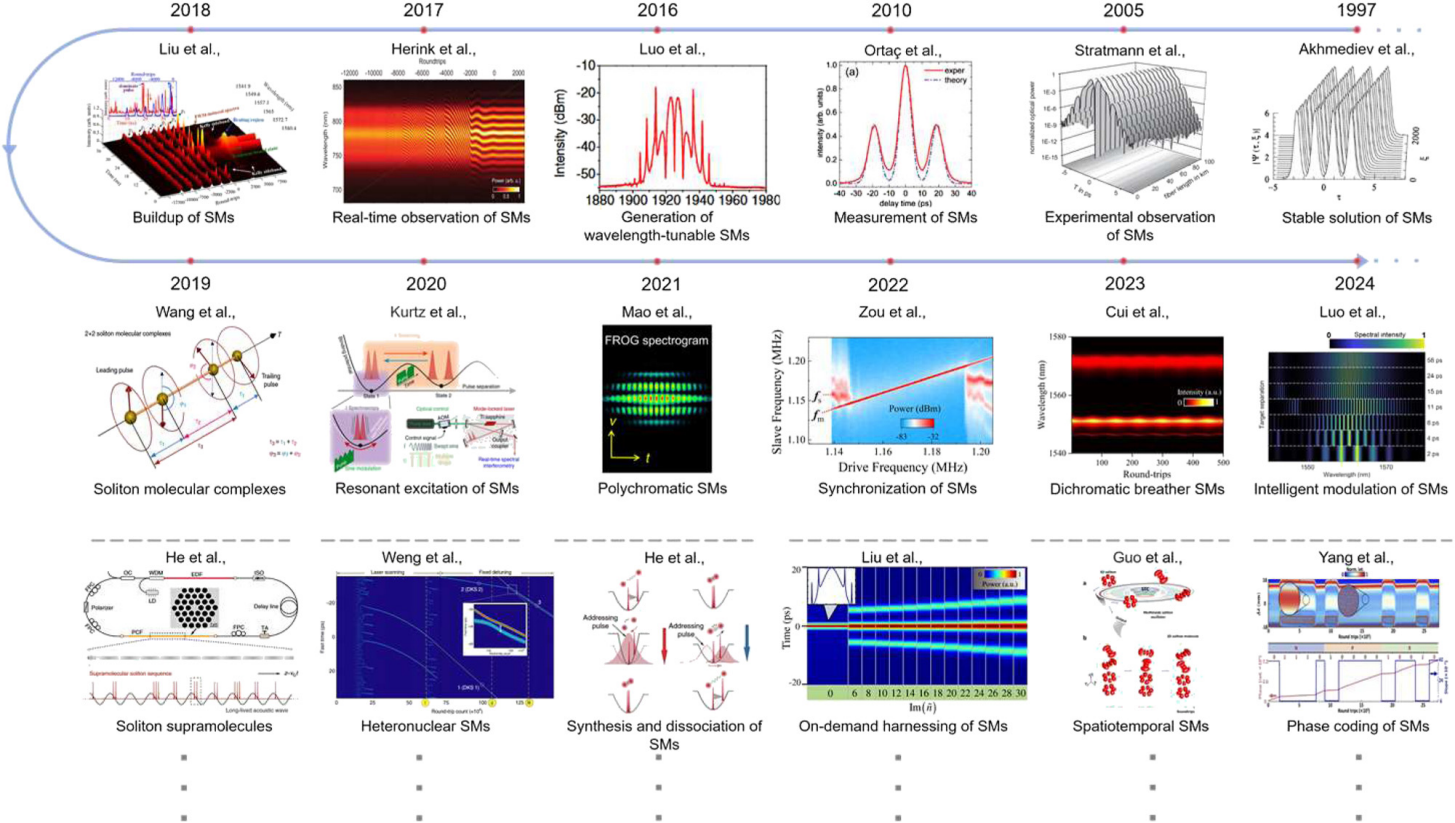
The roadmap for the studies of SMs.

To provide an overview, Figure 1 summarizes the evolution roadmap of SMs with a multidimensional parameter space. The theoretical prediction of SMs was first made using the nonlinear Schrödinger-Ginzburg-Landau model equation [39] and coupled nonlinear Schrödinger equations [40]. Later, analytical and numerical studies were conducted for the identification of the stability of SMs with distinct phase differences between the bound solitons [41], [42], [43]. The first experimental observations of temporal SMs in fibre lasers were reported by the group of Tang [44]. Then, the relevance of soliton molecules to telecommunications was experimentally demonstrated [23]. The autocorrelation technique is often used to measure the pulse separation in SMs [45], [46]. To date, two-soliton bound states have been studied extensively in fibre-laser cavities, in simulations and experiments alike [20], [33], [47], [48], [49], [50]. Numerous experimental observations of SMs have been reported in fibre lasers incorporating mode-locking techniques, such as nonlinear polarization rotation (NPR) [51], [52], [53], nonlinear amplification loop mirrors [54], [55], and various saturable absorbers such as ones using semiconductor saturable absorber mirror [56], carbon nanotubes (CNT) [45], [57], [58], [59], graphene [58], [60], [61], [62], topological insulators [62], [63], black phosphorus [64], VSe2/GO nanocomposite [65], and MXenes [66], [67], [68], [69], [70] (MoS_2_, InSb, chromium sulfide, SnTe, etc.). Beyond the simplest two-soliton bound states, soliton-triplet molecules [71], [72] and even soliton quartets composed of two soliton-pair molecules [73] have also been generated in fibre lasers. Adjusting the polarization state [52] or spectral filter [74] in the laser cavity enables effective wavelength and separation tuning of SMs. Further increase of the pump power can lead to the generation of SM complexes [73] and “supramolecules” [75]. In addition to temporal SMs, manipulating carrier frequency and modal dispersion can yield polychromatic [76], [77] and spatiotemporal SMs [78], [79], and even heteronuclear SMs, formed by incoherently interacting solitons with different carrier frequencies [80], [81]. Beyond those SMs formed by bright solitons, bound states of bright and dark solitons can also be achieved in the zero dispersion regime [82].

Recently, the development of methods of ultrafast temporal optics, such as the dispersive Fourier transform (DFT), time-lens and dispersive temporal interferometer, has made it possible to observe the evolution dynamics of solitons in real time [10], [72], [83], [84], [85], [86], [87], [88]. Using the DFT technique, the entire formation dynamics of SMs in mode-locked fibre lasers has been experimentally revealed [89], [90], offering valuable insights into the complex dynamical behaviour of SMs. Various spatiotemporal dynamical scenarios have been observed in SMs, such as vibrations, breathing, phase slipping, as well as fusion and fission, driven by long- and short-range interaction forces [33], [34]. Meanwhile, nonlinear Fourier transform was also applied to SMs to reveal properties of pure SMs [91]. These findings enhance the understanding of the transient dynamics in SMs, providing deep insight into the complex ultrafast evolution processes in nonlinear optical systems [92].

With the advancement in the studies evolution dynamics of the SMs, a clearer picture of their manipulation mechanisms is emerging, paving the way for the intelligent control of lasing states [93], [94]. For example, experimental studies have shown that SMs exhibit resonance [95] or synchronization [96] in response to external modulation, enabling reversible transitions between different bound states and facilitating all-optical control of soliton–soliton interactions. Meanwhile, various intermediate regimes of SMs, ranging from breathers to chaotic states, have also been explored [97], [98]. Moreover, by adjusting intracavity dispersion parameters, the temporal separation and relative phase of SMs can be selected on-demand, which can be used for quaternary data encoding [99], [100]. Nowadays, SMs can also be intelligently controlled by means of the combination of DFT technology and machine-learning algorithms [94], [101].

In this review, we summarize the state-of-the-art developments for SMs with the multidimensional parameter space in ultrashort fibre lasers. Our focus is on the generation and evolution dynamics of SMs under various conditions. We first present the basic theory for the binding mechanisms and characterization of SMs. Next, we summarize main experimental results for various SM configurations, including different temporal separations, phase differences, pulse numbers, carrier frequencies, and spatial modes. Additionally, we discuss the formation, pulsations, and interaction dynamics of the SMs. Practical strategies for manipulating SMs in the multidimensional parameter space. In this context, the gain modulation, polarization, dispersion management, and photomechanical effects are covered, with applications in data encoding. Finally, in the concluding section we outline unexplored problems, potential challenges, and promising avenues for future research.

## 2 Basic principles of soliton molecules

### 2.1 The basic theory of soliton molecules

For a soliton molecule composed of two identical solitons with a pulse interval of *τ* and a phase difference of *ϕ*, the electric-field envelope of the optical wave in the time domain can be represented as:

(1) $$E\left(t\right)=E_{1}\left(t\right)+E_{2}\left(t\right)=E_{0}\left(t\right)+E_{0}\left(t-\tau \right){\text{e}}^{\text{i}\phi },$$

where complex *E*_0_(*t*) represents the temporal shape of the single soliton, *E*_0_(*t*−*τ*) is its copy shifted by *τ* in time, and *ϕ* is the phase difference between the two solitons.

The spectral amplitude of the SMs is produced by the Fourier transform of the time-domain electric-field envelope (1), which yields

(2) $$E\left(\omega \right)=E_{0}\left(\omega \right)\left(1+{\text{e}}^{-\text{i}\left(\phi -\omega \tau \right)}\right),$$

where *E*_0_(*ω*) is the complex amplitude of the soliton’s spectrum with *ω* being the frequency difference from the carrier optical frequency. This expression shows how the temporal separation *τ* and phase difference *ϕ* affect the spectral characteristics of the two-soliton bound state. Equation (2) gives rise to the expression for the optical intensity of the two-soliton SM:

(3) $$I\left(\omega \right)={\left|E\left(\omega \right)\right|}^{2}=2{\left|E_{0}\left(\omega \right)\right|}^{2}\left(1+\cos\left(\phi -\omega \tau \right)\right).$$

The term cos(*ϕ−ωτ*) in Eq. (3) leads to the modulation in the optical spectrum with period [57]

(4) $$\Delta \nu =\frac{1}{\tau },$$

indicating that the frequency spacing Δ*ν* between adjacent spectral peaks is determined by the time separation *τ* between the bound solitons.

**Figure 2:**
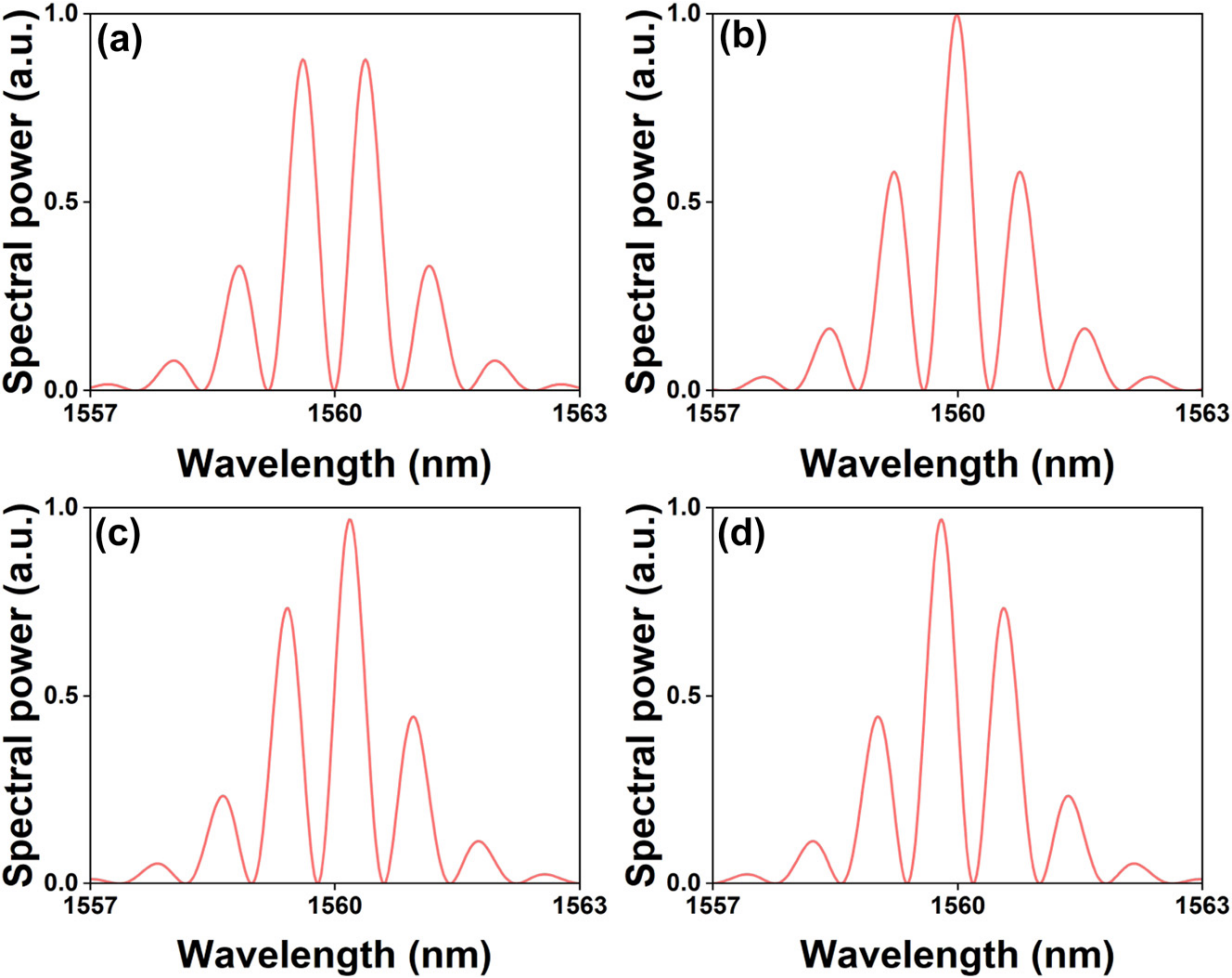
Simulated spectra of two-soliton SMs with *φ* = *π* (a); 0 (b); −*π*/2 (c); and *π*/2 (d), according to Eq. (3). In all figures, 0.8-ps chirp-free, sech-shaped pulses with a central wavelength of 1,560 nm and a temporal separation of 6 ps are used.

When the modulation depth is 100 %, it means that the interference results in complete constructive or destructive interference at different frequencies, depending on the phase shift and the frequency offset [27]. The intensity at the centre of the spectrum (i.e., at *ω* = 0) depends on the phase difference *ϕ*, as shown in Figure 2(a)–(d). For *ϕ* = 0, the two solitons are in phase, resulting in the maximum constructive interference at the centre of the spectrum. For *ϕ* = *π*, the solitons are out of phase, leading to destructive interference and zero intensity at the centre. For *ϕ* = ±*π*/2, the interference results in a spectral shape with intermediate characteristics. The dependence of the spectral intensity on phase shift *ϕ* allows the identification of the relative phase between the solitons by examining the optical spectrum.

**Figure 3:**
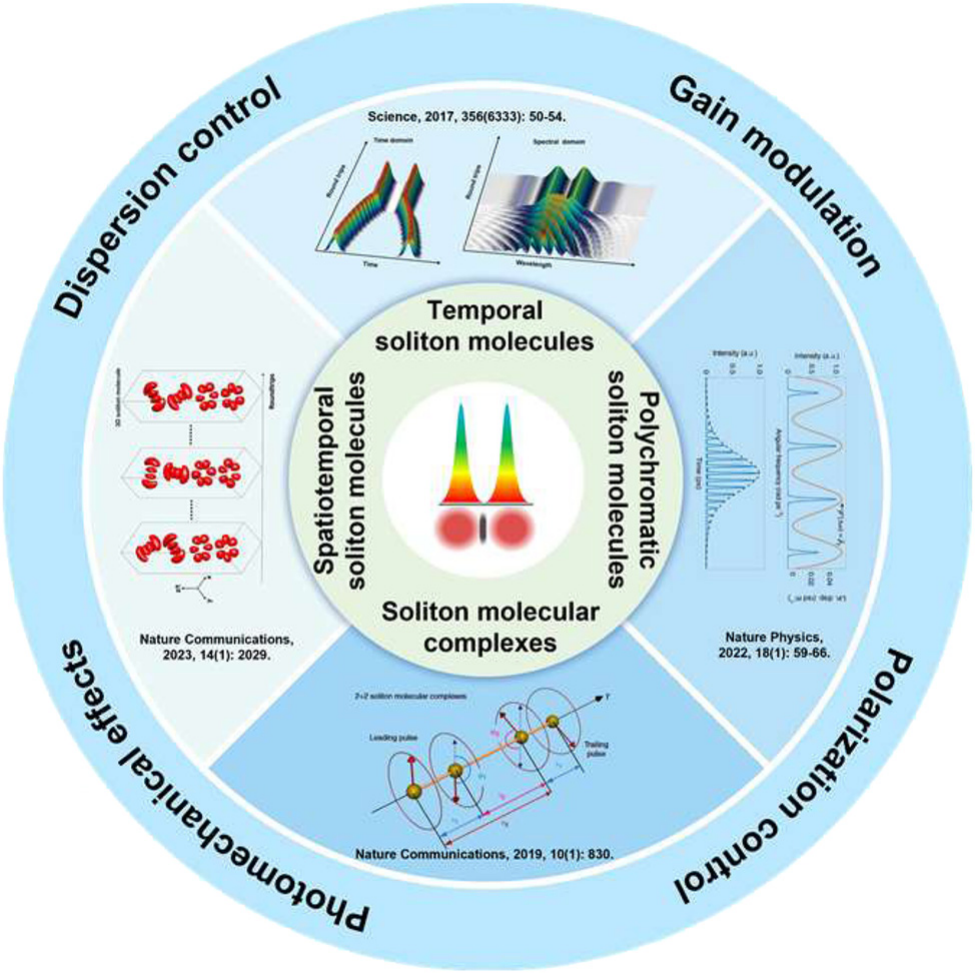
Classification and manipulation of SMs with the multidimensional parameter space.

As shown in Figure 3, SMs can be classified into several types: temporal [89], polychromatic [76], spatiotemporal [79] SMs, and soliton molecular complexes [73]. Temporal SMs consist of a specific number of solitons in the time domain and exhibit interference fringes in the frequency domain. In contrast, polychromatic SMs involve multiple wavelengths in the frequency domain and display interference fringes in the time domain, which is the reverse of the structure features of the ordinary temporal SMs [102]. Spatiotemporal soliton molecules refer to a stable composite state formed by the interaction of multiple spatiotemporal solitons [79], [103]. These molecules exhibit dual localization in both space and time, forming stable structures through nonlinear interactions. This binding mechanism is analogous to the bonding between atoms in molecules, where the interactions lead to a stable and coherent composite entity [104]. As the number of solitons further increases, soliton molecular complexes, alias *supramolecule*s can emerge. Additionally, new types of SMs have been discovered, such as heteronuclear SMs [80], which consist of multiple pulses with distinct carrier frequencies. Another type is represented by SMs in setups with pure-high-even-order dispersion [105]. In typical mode-locked fibre lasers, by adjusting the cavity parameters, one can observe phenomena such as the dynamical behaviour of multi-pulses [106], pulse splitting [107], pulse decay [108], [109], and so on. Such states represent different temporal arrangements of multi-pulse bundles. It can also serve as a fundamental unit, forming harmonic SMs states or soliton bunches [110], [111], [112], [113], [114]. As a result, by precisely controlling the gain through the pump modulation [95], adjusting the loss by the polarization control [115], managing intracavity high-order dispersion [99] and utilizing the photomechanical effect in optical fibres [34] in the laser cavity, the dynamical behaviour of multi-pulse bound states can be finely manipulated, allowing the precise control over the temporal separation, phase difference, pulse number, and other SM characteristics. The precise manipulation of the multi-pulse dynamics offers new possibilities for expanding the control space of multi-pulse systems.

### 2.2 Binding mechanisms of soliton molecules

The formation of SMs following the pulse splitting process in passively mode-locked lasers can be affected by various interactions between solitons in the cavity [92], [116], [117], [118]. Those solitons can also be generated by pulse shaping, resulting in various solitons, including gain-guided solitons, dispersion-managed solitons and vector solitons [119], [120], [121], [122]. Depending on the setup of the fibre laser, one or several interaction mechanisms become stronger than the others and determine the formation and dynamics of the bound solitons. In passively mode-locked fibre lasers, there are a variety of possible interaction mechanisms that relate to different SM modes as follows. First, the gain depletion and recovery effect [123], [124], [125], which arises from the interplay between pulses and the transient behaviour of the gain medium, can stabilize the pulse spacing, particularly in the case of the harmonic mode-locking [126]. Pulses experience the action of repulsive forces due to group-velocity drift, driven by the time-dependent depletion and recovery dynamics in the gain medium [124]. Meanwhile, gain depletion by the pulses can lead to an inhomogeneous noise floor, which can be effectively suppressed by the Casimir effect, leading to long-range attraction [127]. Second, the opto-acoustic effect gives rise to both attraction and repulsion [128], [129], [130], induced in the fibre through electrostriction by high-intensity optical pulses, can create a weak frequency shift between successive pulses. This interaction leads to the pulse-to-pulse attraction, affecting the pulse bunching and stability over the course of multiple round-trips [131], [132]. In fibre lasers employing NPR, a CW component can coexist with solitons and induce their central-frequency shift [133]. When the CW component becomes unstable, solitons develop varying frequency shifts, resulting in different relative velocities. This interaction is linked to motion modes and the harmonic mode-locking [134]. Meanwhile, fundamental solitons interact directly, with the nature of the interaction – attractive or repulsive – depending on their relative phases [11], [135]. Solitons exhibit a repulsive or attractive force if their phase difference is *π* or zero, respectively [136], [137]. These phase-sensitive interactions are most effective when solitons are closely spaced. It should be noted that the phase difference alone does not determine whether the interaction between coherent solitons is attractive or repulsive; rather, the interaction sign depends on the separation between the solitons in an oscillatory manner. Finally, solitons emit dispersive waves due to the periodic action of perturbations in the cavity, such as losses or limited gain bandwidth [11], [138]. The dispersive waves generate localized forces that can either attract or repel solitons, depending on the relative phase and timing between them [139]. The interplay of these mechanisms gives rise to different types of soliton binding.

Based on the binding strength and temporal separation between the solitons, bound states can be broadly classified into tightly and loosely bound SMs [140], [141], alias the ground- and excited-state ones [142]. For tightly bound SMs, local interactions are dominant. The direct soliton–soliton interaction and dispersive-wave interaction contribute to the strong binding of these states, which results in discrete, stable separations between solitons, that define their dynamics and prevent external perturbations from disrupting the bound state. In loosely bound SMs, the solitons are separated by a large temporal distance, which is much greater than their proper widths. As a result, they exhibit weak binding and are susceptible to environmental perturbations. Long-range interactions – such as gain depletion and recovery or acoustic effects – play a dominant role in the formation of such states. The stabilization requires a dynamic balance between competing long-range forces.

In summary, the formation and stabilization of bound solitons depend on a nuanced balance between the long-range and local interaction mechanisms. Loosely bound SMs are dominated by long-range forces, while tightly bound SMs heavily depend on local interactions, leading to distinct dynamics and stability characteristics in the laser cavity.

## 3 Dynamics of soliton molecules

### 3.1 Temporal soliton molecules

#### 3.1.1 Formation dynamics of temporal soliton molecules

The transition from solitons to SMs reflects a deeper understanding of the physics of solitons and signifies essential progress in nonlinear optics research [143]. It was thus discovered that the solitons could exist in optical fibres both as individual modes and in the form of bound states. Bound states of solitons were first theoretically predicted in the framework of the nonlinear Schrödinger–Ginzburg–Landau equation [39] and coupled nonlinear Schrödinger equations [40]. Subsequently, the existence and stability of bound states with phase shits of *π*, 0, and ±*π*/2 were systematically investigated by means of analytical and numerical methods [41], [42], [43].

In 2005, in the context of the study of bound states, Stratmann et al. first coined the term “soliton molecules” to describe the structure of the experimentally observed stable bound states of solitons in a passively mode-locked fibre ring laser [23]. They were observed in the time domain, revealing the stability in the course of the long-distance propagation and suggesting their potential as a new data-encoding method to enhance the bit rate of fibre-optic communications. Subsequently, Grelu et al. elaborated on the SM concept of SMs [144], construing them as stable structures formed by the interaction of solitons in the nonlinear medium. Since then, SMs have been widely found in various fibre lasers with different dispersion and mode-locking regimes.

**Figure 4:**
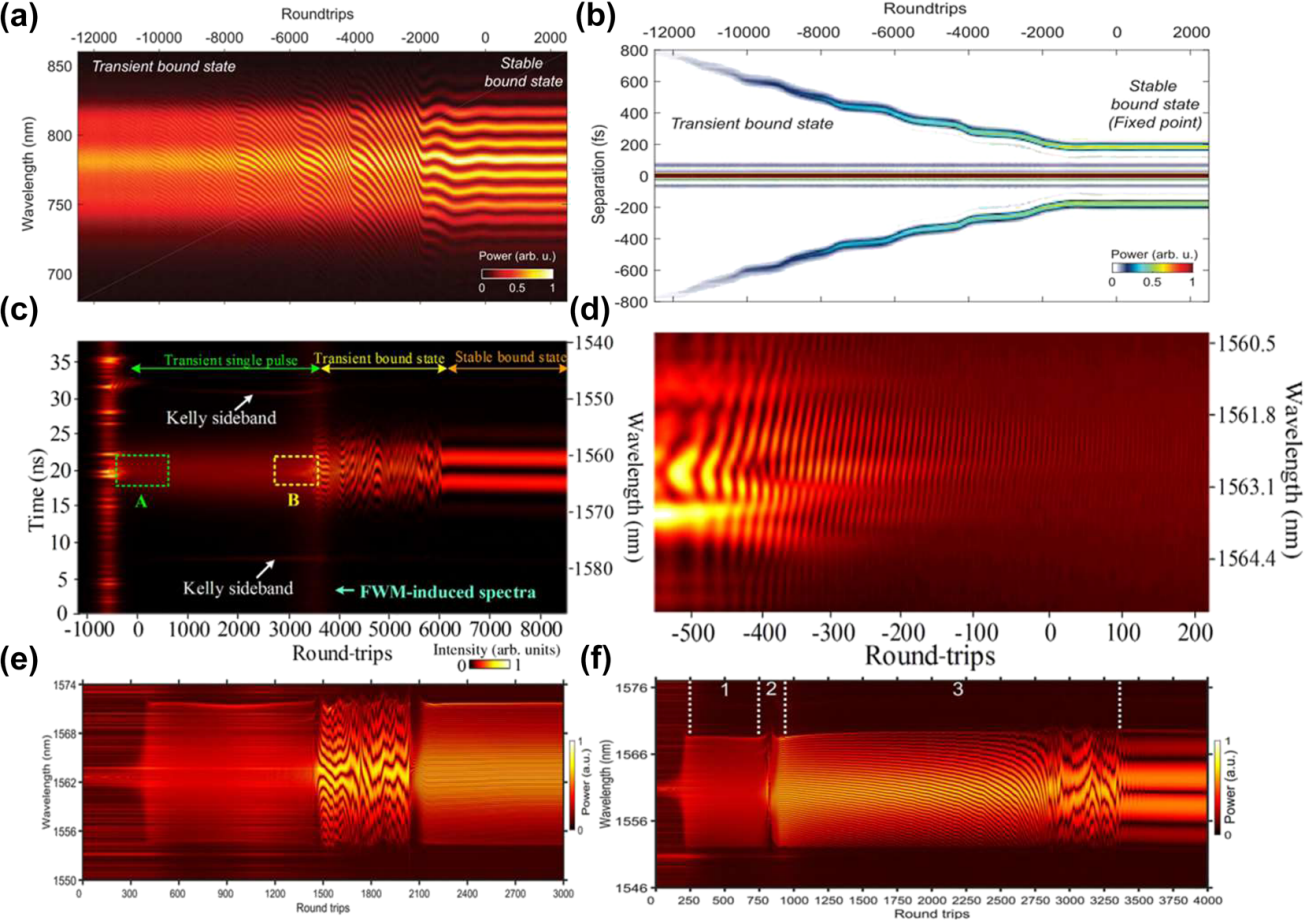
Various scenarios of the formation and evolution dynamics of SMs. (a, b) The formation of SMs from a transient bound state and corresponding field autocorrelations (Herink et al. [89] © AAAS 2017). (c, d) The formation and evolution of SMs featuring beating dynamics (Liu et al. [90] © American Physical Society 2018). (e) The formation dynamics of ground-state SMs; (f) the build-up dynamics of excited-state SMs (Peng et al. [142] © Wiley-VCH Verlag 2018).

The dynamics of SMs had not been fully elucidated in experimental studies until 2017. Constrained by the scanning speed of detection devices, research into complex transient phenomena relied predominantly on high-speed oscilloscopes for time-domain monitoring and lacked experimental observation of the real-time spectral evolution. With the rapid advancement of real-time-measurement technologies, Herink et al. employed the DFT technique [89], which maps pulse spectra to temporal waveforms [145], [146], [147], to acquire real-time spectral information. They were the first to investigate the spectral evolution of femtosecond SMs within a few cycles of a mode-locked laser cavity, as shown in Figure 4(a) and (b), tracking two- and tri-soliton SMs over hundreds of thousands of consecutive cavity roundtrips. The measured results identified fixed points and periodic as well as aperiodic SMs orbits and revealed various complex transient dynamics scenarios in ultrafast lasers. Thus, the DFT technique has enabled experimental observation of complex transient multi-soliton dynamics in mode-locked fibre lasers, including soliton interaction, soliton collision, and soliton explosions [33], [90], [148]. Compared to conventional spectrometers, the DFT technique offers the additional benefit of the dispersive medium also functioning as an optical amplifier, optimizing the balance between the sensitivity, speed, and resolution through the distributed amplification of optical pulses in the dispersive fibres [106], [148], [149]. Subsequently, the DFT technique has been extensively employed by researchers to observe and analyse complex soliton dynamics, paving the way for real-time observations of interactions in complex nonlinear systems [150], [151], [152].

With advancements in real-time detection technology, the complete build-up process of establishing stable, long-lived SMs, akin to single solitons [153], [154], [155], has been elucidated. By minimizing external disturbances and optimizing the laser system, Liu et al. successfully tracked the formation and evolution of SMs in a CNT-based mode-locked fibre laser [90]. They have demonstrated that the formation dynamics of stable SMs proceed through five distinct stages, as illustrated in Figure 4(c): the raised relaxation-oscillation (RO) stage, which is a characteristic feature of the transient behaviour in lasers [154], the beating-dynamics stage, as shown in Figure 4(d), the transient single-pulse stage, the transient bound state, and, finally, the stable SMs. Furthermore, Peng et al. demonstrated the formation of ground- and excited-state SMs [142]. In both cases, the formation phase involves three nonlinear stages: mode-locking, soliton splitting, and soliton interaction. For ground-state SMs, repulsive interactions prevail, as displayed in Figure 4(e). For excited-state SMs, as shown in Figure 4(f), the interactions exhibit a wide range of behaviours in repeated measurements, including attraction, repulsion, collisions, vibrations, and annihilation. Subsequently, Zhou et al. reaffirmed the formation process of SMs in the normal-dispersion regime [47]. They demonstrated that a single soliton initially forms from noise in the time domain, and subsequently splits into two solitons, which then interact with each other. Ultimately, stable sub-molecules with well-defined pulse separations are formed.

The dynamics of SMs has sparked widespread research interest. In 2022, Du et al. reported a comprehensive study of the binding mechanism and internal dynamics of asymmetric bound states composed of a weak and strong solitons with the corresponding short-range interactions [156]. They found that, in this case, the solitons achieve a balanced separation and energy distribution through periodic energy exchange, mediated by overlapping tails. In parallel, Zhang et al. have integrated a time-lens system with the DFT technology to elucidate the spectral-temporal characteristics of dissipative SMs in a passively mode-locked erbium-doped fibre laser, revealing various internal dynamical phenomena, such as attraction, creeping, period-doubling breathing, and collisions [157]. This discovery represents a significant advancement in the studies of soliton interactions. As the standard silica-based optical fibres generally feature strong anomalous chromatic dispersion at 2 μm, various SMs can also be generated by injecting intense pumping [52], [158], [159], [160]. Employing a linearly chirped fibre Bragg grating as a dispersive element in DFT measurements, the direct real-time observation of SMs in the mode-locked fibre laser operating at wavelength 2 μm has been achieved [161]. Furthermore, by controlling the intracavity group delay between polarization modes, two orthogonally-polarized dissimilar pulses can be trapped, as a vector mode, in a mode-locked Tm-doped fibre laser, thus resulting in the formation of dissimilar SMs [162]. This novel bound state features trapping between two ultrafast vector pulses with distinct properties including the energy, duration, and chirp, leading to unique temporal and spectral profiles. This series of experimental achievements reflects the ongoing deepening and broadening of soliton research in recent years.

#### 3.1.2 Pulsation dynamics of temporal soliton molecules

SMs show diverse dynamical behaviours, including vibrations or pulsations, i.e., oscillatory pulse structures [163], [164], [165]. Vibration may be considered as a broader form of pulsations, encompassing a wider range of oscillatory behaviours. This dynamical regime is important for understanding complex wave interactions in nonlinear media. The pulsation dynamics refer to the oscillations that arise in the course of propagation due to external disturbances or internal nonlinear interactions [164], [166]. As early as in 2006, vibrational dynamics of SMs in ultrafast lasers were predicted [167], but real-time experimental validation was initially hindered by measurement limitations. Recently, with the advancements in real-time measurement techniques, the DFT technique has become a standard for observing soliton dynamics in real time, advancing, in particular, the studies of the SM pulsations. As mentioned above, the key factors which determine the SM dynamics are the temporal separation and phase shift between the bound solitons [168].

**Figure 5:**
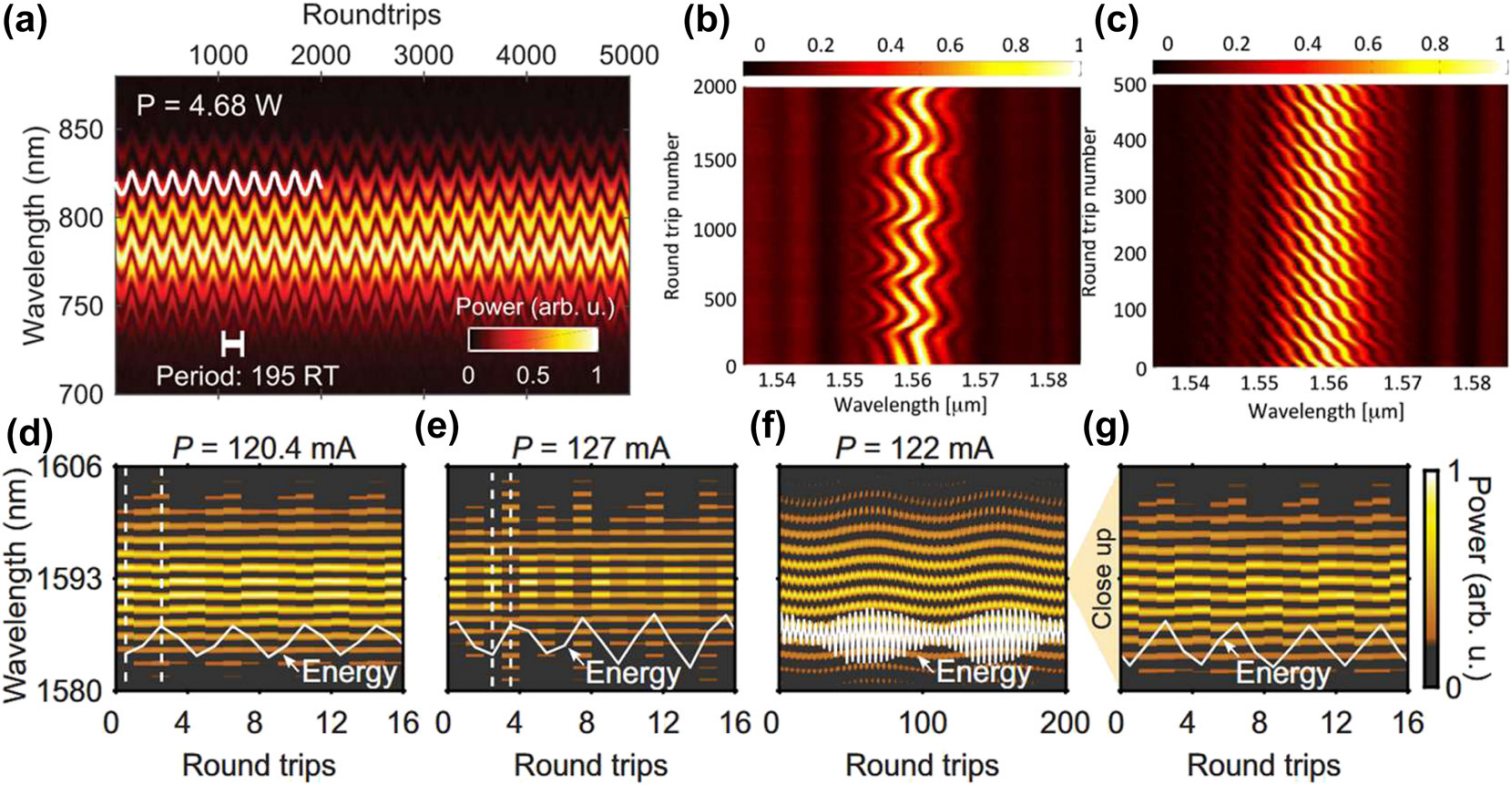
Various dynamical scenarios of pulsation SMs. (a) SM featuring an oscillating time separation and phase (Herink et al. [89] © AAAS 2017). (b, c) SM featuring vibration and sliding phases (Krupa et al. [33] © American Physical Society 2017). (d–g) The experimental observation of subharmonic, modulated subharmonic, and non-subharmonic diatomic breathing SMs (Wu et al. [97] © American Physical Society 2023).

In 2017, Herink et al. used the DFT technique to study the internal dynamics of SMs in real time [89], observing both periodic and non-periodic oscillations caused by nonlinear phase delays in the gain medium. These delays caused dynamical changes in the solitons’ relative phase and separation. They also suggested that the vibrational behaviour might be linked to topological protection, with some excitations exhibiting topologically protected characteristics. Figure 5(a) illustrates the typical spectral evolution of SMs oscillations, dominated by relative phase oscillations. Meanwhile, Krupa et al. identified two types of pulsations: one where both relative phase and pulse separation oscillate together (similar to vibrations of a diatomic molecule) and another with only phase slipping [33], as shown in Figure 5(b) and (c). In 2018, Peng et al. introduced a conceptually distinct type of SMs, known as intermittently vibrating SMs that demonstrate a transition between vibrating and static states [142]. Wang et al. used the DFT technique to show that SM pulsations synchronize with the periodic appearance of Kelly sidebands [73], i.e., dispersive waves emitted due to the interplay of nonlinear losses and cavity components [169]. In 2019, Chen et al. found that SMs can feature simultaneous pulsations in their energy, separation, and phase shift. In double SMs, the separation and phase pulsate synchronously with each round-trip, showing either regular or irregular behaviour [170]. They also observed similar dynamics in triple soliton molecules. Regular pulsations occur when the phase and separation remain consistent through each cycle, while irregular pulsations happen when these states vary [170]. This indicates that mutual locking or periodic evolution of pulse separation and phase difference is crucial for achieving regular pulsations in SMs. Besides, symmetric or asymmetric distortions in their profiles and energy-exchange processes can also be observed [168]. Peng et al. have experimentally confirmed the existence of breathing SMs in the normal-dispersion mode-locked fibre lasers, overcoming the prior limitation of their confinement to micro-resonator platforms [171]. The following year, more complex dynamics were discovered, Xia et al. discovered, beyond the simple oscillatory dynamics, two types of oscillating soliton pairs exhibiting quasiperiodic and chaotic phase oscillations, respectively [172]. These complex internal dynamics are governed by subtle energy flows between components, affected by the gain dynamics and soliton interactions.

In 2022, Hamdi et al. successfully applied the super-localization technique to observe the external motion of SMs in a fibre ring-cavity laser in the time domain, showing that oscillating SMs, separated by a time delay of several nanoseconds, can be synchronized [173]. Additionally, Zou et al. have experimentally confirmed the synchronization of internal vibrations of self-excited oscillating SMs by injecting modulation signals into the laser cavity [96]. They scanned for rational multiples of the SMs’ free vibrational frequency and used the high-precision balanced optical cross-correlation (BOC) to monitor real-time responses. They discovered sequences of locked states where the vibrational frequency was controlled by the injected signal, following the Arnold’s-tongue model. These results have demonstrated efficient synchronization across subharmonics, fundamental harmonics, and superharmonics, as displayed in Figure 5(d)–(g). In 2023, Wu et al. further investigated the synchronization in breathing SMs [97], showing that they can be synchronized with the laser-cavity’s second harmonic frequency. During desynchronization, hysteresis effects were observed between the internal components of the molecule and an intermediate state, characterized by subharmonic breathings and anharmonic sidebands, was identified. They also observed chaotic SMs in an ultrafast fibre laser, using BOC, to measure relative optical-pulse intervals with sub-femtosecond precision in real time [96]. By injecting modulated optical signals, they have demonstrated a possibility to switch between ordered and chaotic states, thus enabling the optical control of SMs and paving the way for soliton-based logic gates and chaotic communications. Zou et al. later expanded this research by observing quasi-periodic behaviour and transitions to chaos in SMs, showing intrinsic frequency locking and its independence from laser repetition rates [174]. By means of a simultaneous time-frequency analysis, the phase-diagram analysis and Lyapunov-exponent analysis, they were able to demonstrate the connection between the quasi-periodic scenario and chaotic dynamics.

Recent studies have also focused on pure-quartic solitons (PQSs), with findings indicating that PQS pulse energy is proportional to the third power of the inverse pulse duration [175], [176], [177]. In this context, Yang et al. have explored numerically the internal dynamics of pulsating PQS molecules associated with the fourth-order dispersion in mode-locked fibre lasers [178]. Traditional soliton pulsations involve periodic energy fluctuations in time and frequency domains, while a new phenomenon, termed “invisible” soliton pulsations [179], was discovered using real-time spectral measurement. This phenomenon involves periodic changes in the spectral profile and peak power without the respective changes in the pulse’s energy. In 2024, Yan et al. explored pulsation dynamics in Mamyshev oscillators, identifying both “visible” and partially “invisible” dynamical regimes in symmetric and asymmetric SMs [180]. “Invisible” pulsations were attributed to intensity differences between the bound pulses. These studies offer new insights into the complex dynamics of optical SMs, providing a basis for further research and development of potential applications.

#### 3.1.3 Interactions of temporal soliton molecules

Soliton interactions are a crucially important topic in nonlinear dynamics, particularly in understanding the behaviour of solitons in fibre laser systems [41], [181], [182]. The interactions involving SMs exhibit complex dynamical characteristics, including the energy exchange, spectral modulations, and variation in the temporal separation between the bound solitons [48].

**Figure 6:**
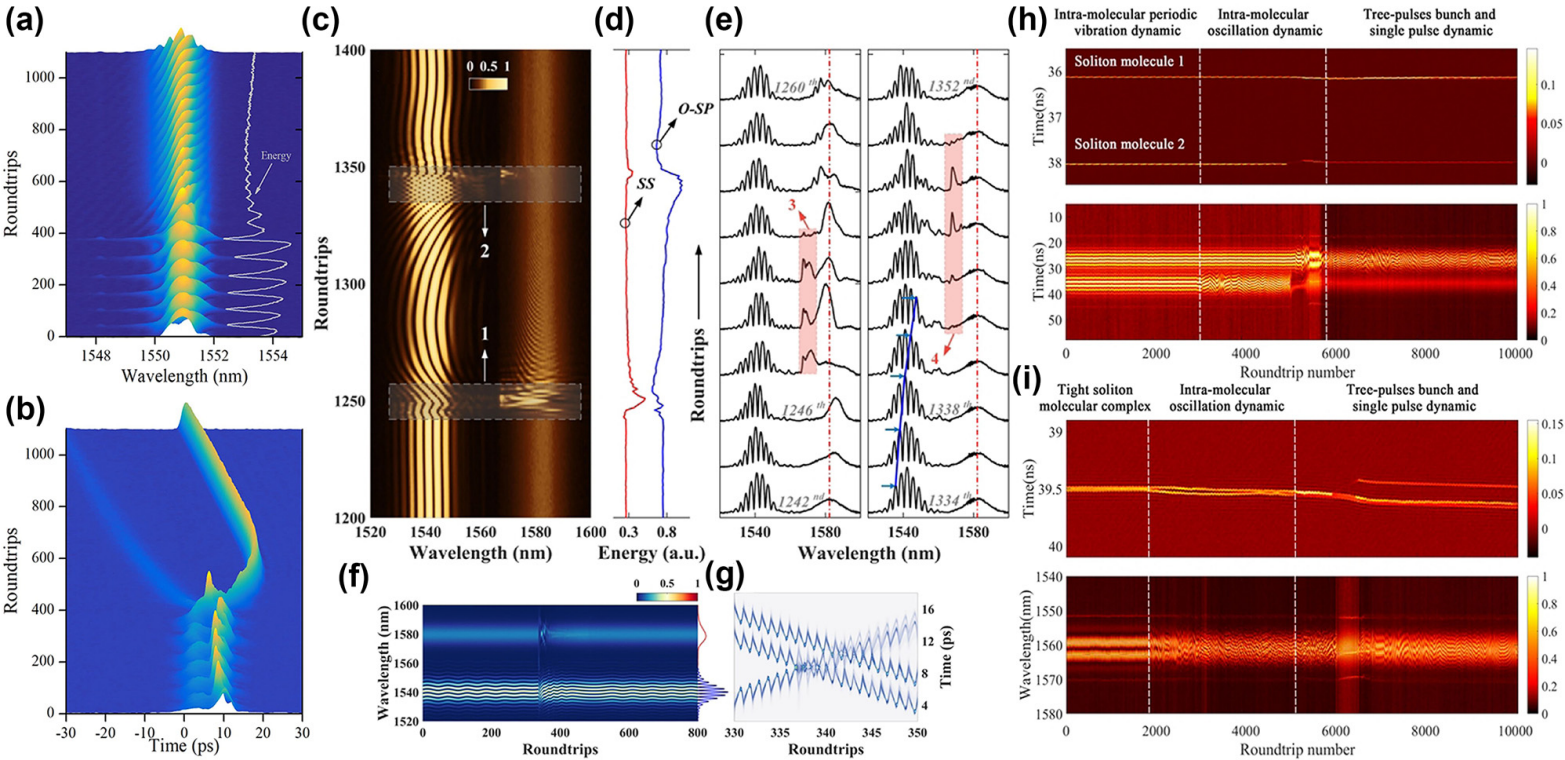
Various scenarios of the SM interactions. (a, b) The formation of a dissipative SM by colliding free solitons (Zhang et al. [157] © Elsevier Ltd 2022). (c–g) Close-up of the collision, the subsequent partial spectral collapse of SM and numerical simulations of the collision between the SM and a free soliton (Liu et al. [183] © The Optical Society 2024). (h, i) The indirect and direct interactions of SMs (He et al. [184] © The Optical Society 2023).

Recent studies have shed light on the intricate behaviours of SMs. In particular, it was highlighted how dispersive waves generated during the collision of two single solitons affect their interaction, leading to the formation of new SMs with variable separations, as shown in Figure 6(a) and (b) [157]. These findings suggest that the increase of the separation causes the solitons to repel each other, leading to a smaller group velocity of the newly formed soliton SMs. Liu et al. expanded these insights by examining collisions between an SM and a free soliton in dual-wavelength mode-locked fibre lasers [183]. Their observations indicated that energy accumulation during collisions leads to an explosive behaviour of the SMs, mirroring the dynamics observed in collisions of material particles. The results displayed in Figure 6(b)–(g) demonstrate spectral repulsion and energy exchange between the colliding SM and free soliton, emphasizing the delicate balance of the attraction and repulsion forces.

The dynamics of SMs was further explored by He et al., revealing that gain perturbations can induce oscillations and collisions between the pulses of which the SMs are built [184]. That work identified distinct splitting pathways for SMs based on their initial separations, demonstrating a complex progression through multiple stages, including periodic vibrations and transitions into three-pulse complexes and single pulses. Figure 6(h) and (i) present several typical spectra illustrating the indirect and direct interaction processes of SMs. The results show that oscillation and collision behaviours of pulses within SMs can be induced by gain perturbation, along with pulse recombination through interactions between SMs. Overall, the dynamics of the interactions including SMs highlight a rich variety of scenarios. The collision processes include the disintegration and rebuilding of SMs as well as chaotic oscillating evolution [185], accompanied by the emergence of transition states such as triple binding state, soliton fusion and acceleration. These findings not only expand the understanding of soliton dynamics but also offer new possibilities for applications in nonlinear optical systems, such as enhancing data-processing capabilities and the development of advanced laser technologies. Future research is expected to further reveal the complexity of the soliton dynamics in fibre lasers and related potential applications.

### 3.2 Polychromatic soliton molecules

**Figure 7:**
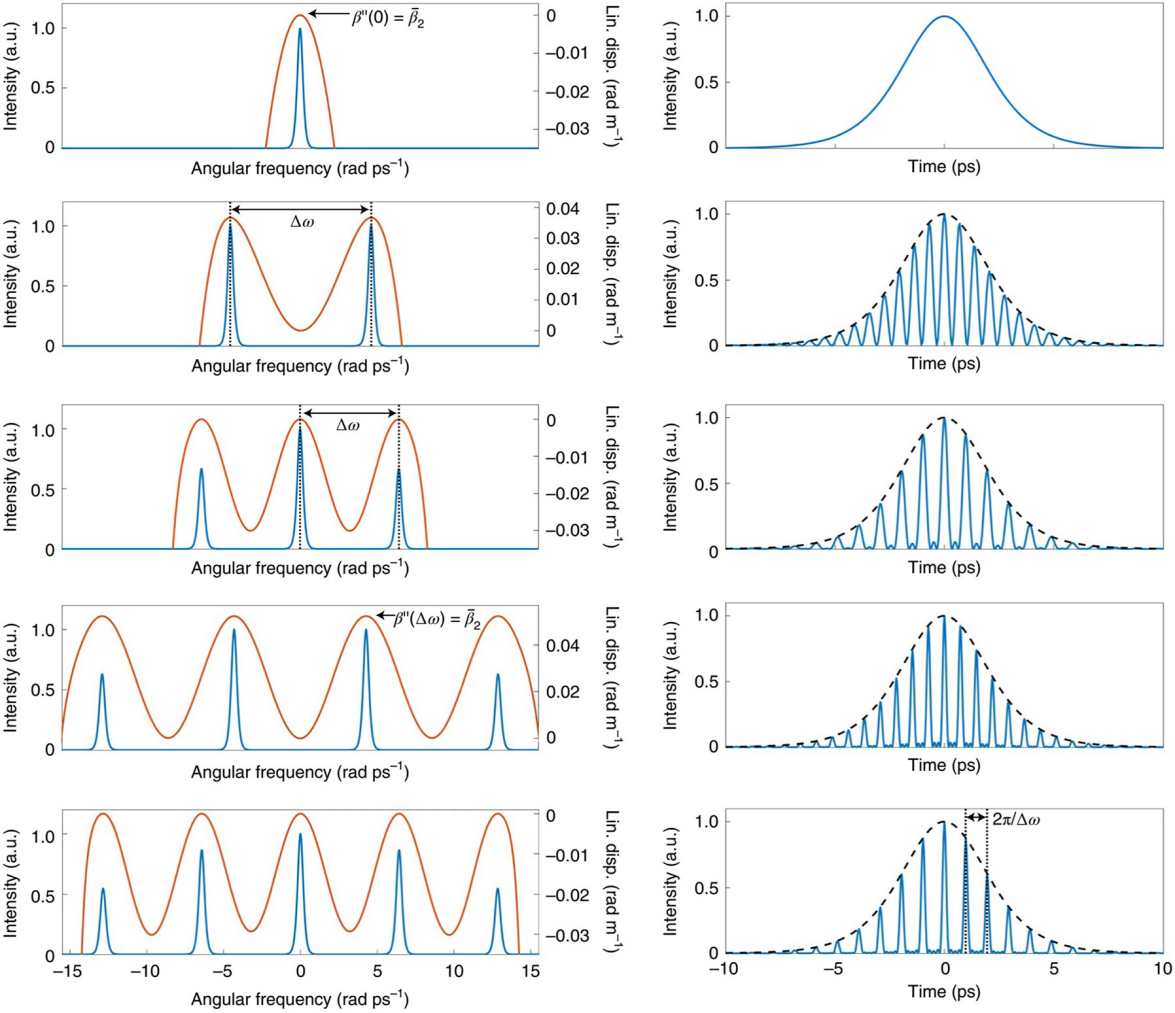
Linear dispersion relations and the corresponding numerical solutions for polychromatic SMs, as per Ref. [76] (© Springer Nature 2022).

**Figure 8:**
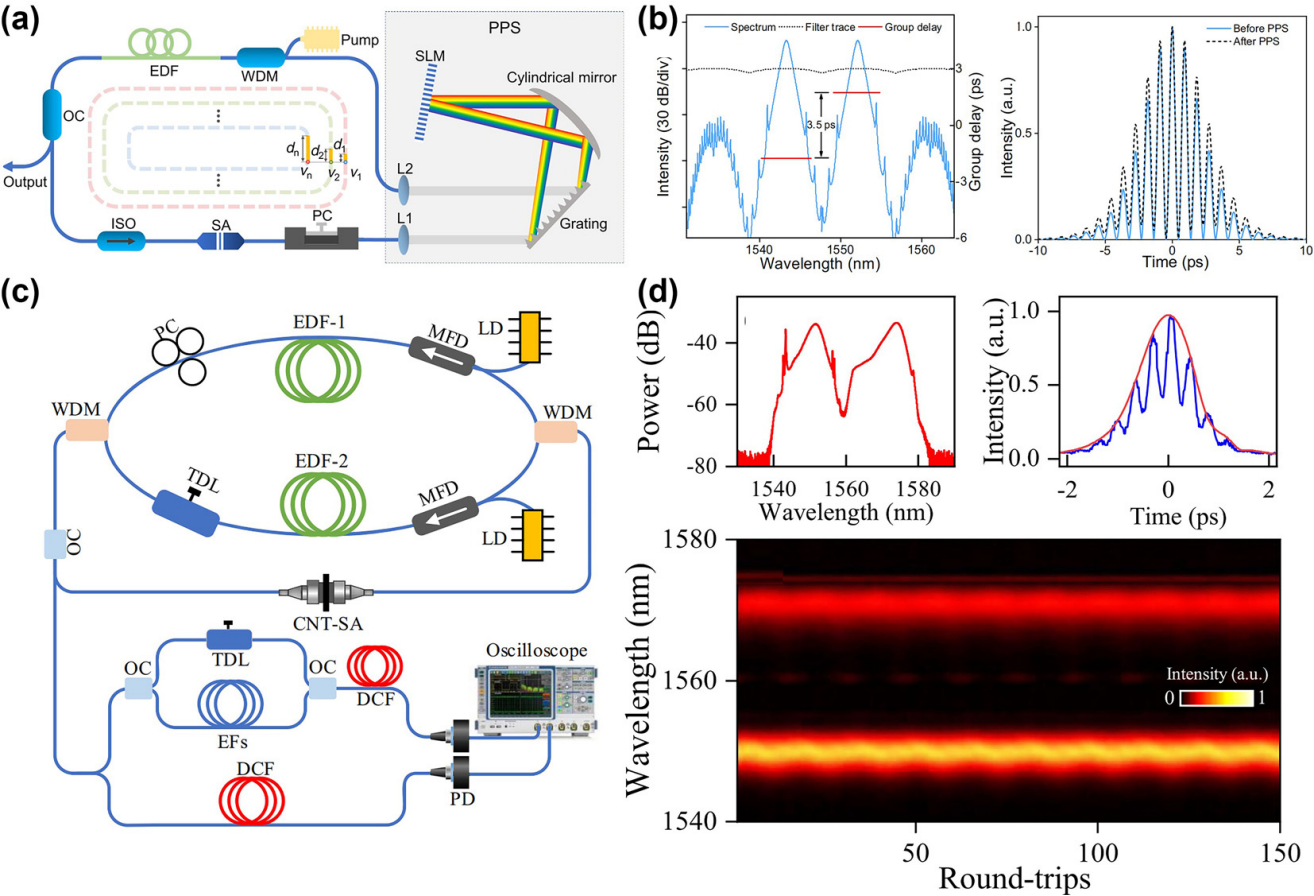
Two different mechanisms for the formation of dichromatic SMs: (a, b) The experimental setup and output spectra for the formation of dichromatic SMs through the dispersion management (Mao et al. [77] © Springer Nature 2021). (c, d) The experimental setup and output spectra for the formation of dichromatic SMs by the XPM effect (Cui et al. [192] © American Physical Society 2023).

In polychromatic SMs, each soliton can have a different central wavelength, but they are synchronized in time and maintain a fixed phase relation. The formation of such SMs is typically determined by the use of dispersion management [186] in the cavity and characteristics of the nonlinear medium [187]. In 2019, Melchert et al. predicted the formation mechanism of SMs with two frequencies, along with their characteristics and potential applications, drawing a striking analogy to quantum mechanics [188]. Lately, Lourdesamy et al. pointed out that the intracavity dispersion is an important factor affecting the formation of polychromatic SMs [76]. As shown in Figure 7, under the guidance of the positive second-order and negative fourth-order dispersions in the cavity, the dispersion relation exhibits a dual-peak shape, being symmetric about the central frequency. As the positive second-order dispersion coefficient increases, the spectral separation between the two solitons increases as well, forming two distinct peaks in the spectrum, which are located at the two peak values of the dispersion relation. In the time domain, they gradually reveal a clear hyperbolic-secant shape. This dual-peak structure of the dispersion relation is a key feature supporting the formation of polychromatic SMs. Meanwhile, they experimentally demonstrated a nonlinear enhancement factor of up to 3.5 in a mode-locked fibre laser incorporating an intra-cavity spectral pulse shaper, with the potential for achieving even higher enhancements. Based on the management of intracavity dispersion, Mao et al. have demonstrated a method to achieve synchronization of solitons with different carrier wavelengths by mode-locking using intracavity group-delay modulations [77]. Figure 8(a) and (b) show the experimental setup and output spectrum in frequency and the time domains. By utilizing a programmable pulse shaper (PPS) to control the intracavity group delay, the synchronization of sets composed of two to five solitons with different carrier wavelengths was realized. By introducing convex-concave frequency phases [81], they were also able to obtain heteronuclear polychromatic soliton compounds, composed of chirp-free conventional solitons and chirped dissipative solitons. Meanwhile, these polychromatic fibre laser exhibits Talbot self-imaging effect in the breathing state [189].

In addition to changing the intracavity dispersion to produce polychromatic SMs, cross-phase modulation (XPM) in a common saturable absorber is also one of the key mechanisms [190], [191]. In 2023, Cui et al. reported dichromatic SMs by designing a passively synchronized dual-wavelength mode-locked fibre laser [192]. The experimental setup and output spectra of the dichromatic SMs are shown in Figure 8(c) and (d), respectively. This fibre laser provides the visibility of the breathing dynamics of dichromatic SMs in the time-frequency domain through DFT and dispersion-delayed self-interference techniques. The XPM-based method provides new possibilities for the simulation of SMs in optical systems with a multidimensional parameter space and is of significant importance for the design and applications of ultra-short pulse lasers [193], [194]. By further optimizing the pump power, both static dichromatic SM compounds – as hybrid bound states of multiple soliton pulses – and those with robust internal vibrations can also be achieved [195].

When solitons exhibit different characteristics, such as spectrum and intensity, the resulting polychromatic SMs can be regarded as “heteronuclear” ones. Melchert et al. [80] theoretically studied heteronuclear SMs formed by the strong binding force between solitons with different central frequencies through the Kerr nonlinear effect. Kippenberg et al. generated heteronuclear SMs in the same magnesium fluoride whispering-gallery-mode microcavity, composed of cavity solitons with different amplitudes, widths, and carrier frequencies [196]. These states are not only significant for understanding the soliton dynamics in complex nonlinear systems but also enable the generation of coherent frequency combs. Additionally, Del’Haye et al. experimentally produced a heteronuclear bound state in a microcavity, formed as the combination of bright and dark solitons [197]. In a near-zero-dispersion fibre laser, because of the incoherent cross-phase modulation of light, “polyatomic” SMs that are able to bind with bright and dark solitons can also be created [82]. The study of polychromatic SMs contributes a deeper understanding of the dynamical behaviour of SMs and holds the promise for advancing applications of related technologies to optical communications, fibre sensing, and precision measurements. For instance, the specific spacing and phase characteristics of the polychromatic SMs can be utilized to achieve more efficient data transmission and more accurate measurements [198]. Additionally, polychromatic SMs have attracted significant interest for their potential applications in attosecond laser generation, THz lasers, ultrafast nonlinear optics, and related fields [76], [77].

### 3.3 Spatiotemporal soliton molecules

The spatiotemporal solitons are high-dimensional localized waves generated in multi-mode fibre lasers by precisely balancing the modal dispersion with nonlinear effects and synchronizing the transverse and longitudinal modes. Along with the balance between the gain and loss, this mechanism supports spatiotemporal dissipative solitons (STDS) [199], [200]. For temporal solitons, the mode-locking in time relies on the nonlinear effects and dispersion management in the cavity to achieve pulse compression and stabilization. In contrast, spatiotemporal mode-locking (STML), in addition to the nonlinearity and dispersion, also makes use of the spatial gain distribution, modal dispersion, spatial filters, and other spatial-dimension effects, which collectively influence the synchronization of transverse and longitudinal modes [201]. In this context, the dispersion encompasses not only the chromatic (intramodal) dispersion, but also the intermodal dispersion, which refers to the phenomenon in which different transverse modes propagate at different group velocities in multimode fibres, causing the transverse modes to gradually separate and diverge. How to balance the dispersion in multimode fibre lasers, including the intramodal and, especially, intermodal dispersions, is an important issue for the realization of STDS [201]. It was first achieved in normally dispersion multimode fibre lasers [103]. Then, Wu et al. conducted the first detailed study on the spatiotemporal mode-locking in the laser with anomalous dispersion. Different mechanisms, such as the Kerr nonlinearity, spatiotemporal saturable absorbers, and spatial coupling/filters, may be used for the compensation of the intermodal dispersion, which is crucially important for the understanding of the nonlinear dynamics in spatiotemporal mode-locked lasers and optimization of the laser design. Multimode fibre lasers offer a flexible platform for achieving STML. The expansion into the spatial dimension introduces complex nonlinear spatiotemporal interactions and a wealth of spatiotemporal physical phenomena [202].

**Figure 9:**
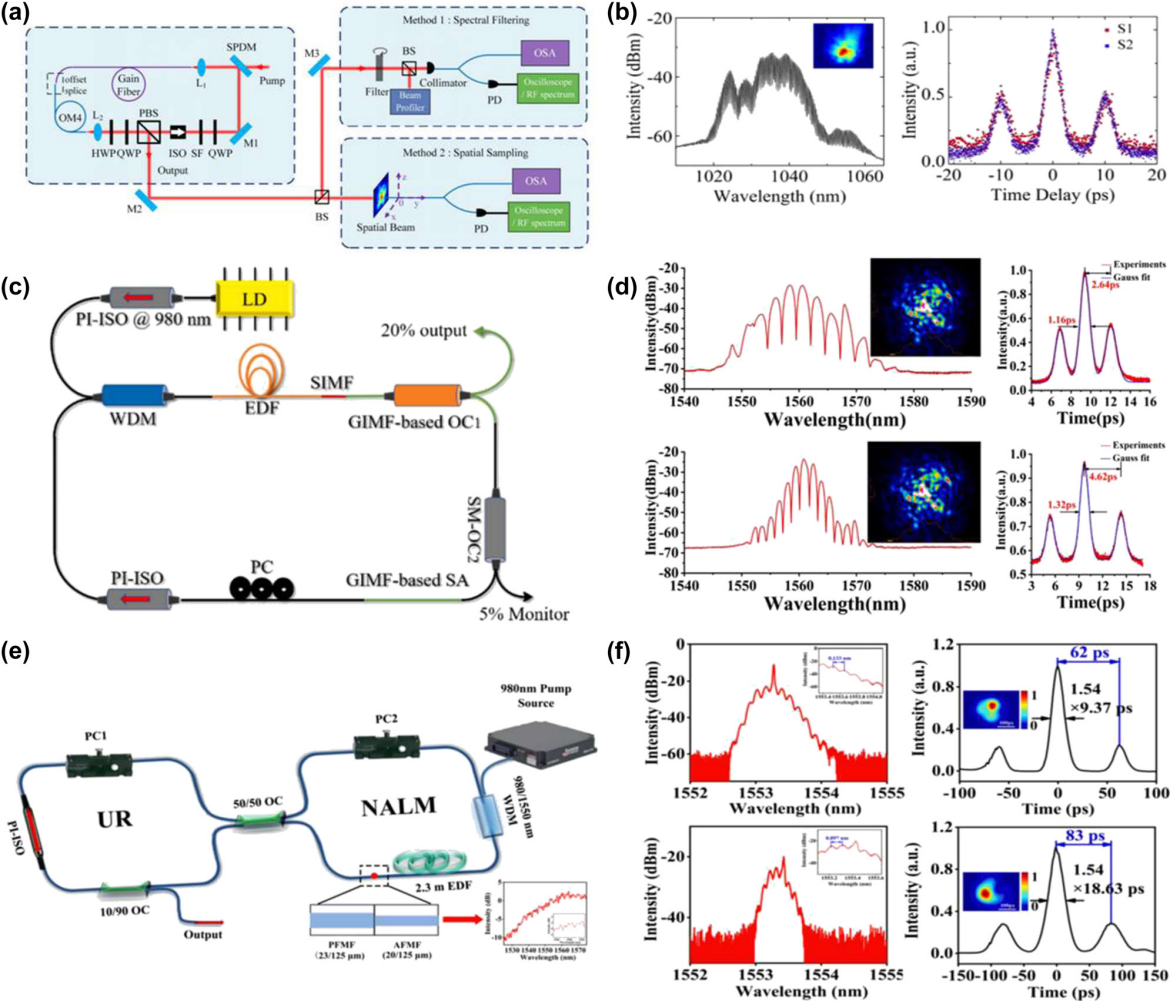
The experimental setup and spectral output characteristics for different cavity structures. (a, b) The formation of STSMs in the annular cavity, including a fully multimode fibre (Ding et al. [204] © The Optical Society 2019). (c, d) The formation of STSMs in the all-fibre hybrid annular cavity (Guo et al. [205] © Elsevier Ltd 2022). (e, f) The formation of STSMs in the ring cavity, including a few-mode fibre (Wu et al. [206] © Elsevier Ltd 2022.

In a fully multimode fibre cavity, the formation of (STSM) depends on the intracavity nonlinear effects and dispersion management. Yang et al. firstly observed the STSM in a spatiotemporal mode-locked multimode fiber based on the NPR technique [203]. By adjusting the pump power and using intracavity elements such as wave plates, the appropriate energy exchange and mode locking between different transverse modes can be achieved. Various SMs, including soliton pairs, soliton triplets, and soliton quartets with different pulse separations, have been achieved. As shown in Figure 9(a) and (b), Ding et al., working with a ring cavity based on a multimode fibre, have successfully initiated and controlled the formation of soliton molecules under multimode conditions [204]. The harmonic mode-locking and multi-pulse phenomena in the multimode fibre laser have been observed in the experiment. Through the experimental observations and numerical simulations, the nonlinear dynamics related to these phenomena were analysed, and the impact of cavity parameters on the spatiotemporal output was identified. In a hybrid cavity with both single-mode and multimode components, the formation of STSM involves the interplay between sections of single-mode and multimode fibres. As illustrated in the Figure 9(c) and (d), Guo et al. have constructed a novel all-fibre hybrid ring-cavity structure, which operates in the STML mode, integrating single-mode and multimode fibre segments [205]. In the hybrid cavity, longitudinal and transverse mode locking is achieved by adjusting the polarization controller and multimode fibre segment, respectively. Adjusting the polarization controller, loosely and tightly bound soliton pairs were generated at the carrier wavelength 1,560 nm. A few-mode fibre-laser cavity typically has a limited number of transverse modes, which is nevertheless sufficient to support the formation of spatiotemporal SMs. Using an annular cavity based on the full multimode fibre, Wu et al., for the first time, have achieved the switching generation of femtosecond and picosecond STML pulses in a few-mode fibre figure-of-eight laser at carrier wavelength 1.55 µm [206]. As shown in Figure 9(e) and (f), by appropriately adjusting the pump power or polarization controller, STSM pulses with different widths, including single and multiple pulses and SMs, can be generated. Different cavity structures provide a variety of physical conditions and means of control, making the generation and regulation of STSM diverse and flexible [207]. By precisely controlling the intracavity nonlinear effects, dispersion, gain, and loss, the stable generation and flexible control of STSMs can be achieved in different cavity structures.

The motions of STSMs typically begin at unpredictable times (i.e., they are largely non-repeatable), and their subsequent evolution can last for several milliseconds. Time-averaged studies of STSMs have been demonstrated [203], [208]. Using single-shot multispeckle spectral-temporal measurement technology, which leverages optical time-division multiplexing and DFT, Yang et al. enabled the simultaneous observation of multiple speckle grains, providing insights into the long-term evolutionary dynamics of STDSs [78]. Under the spatiotemporal coupling effect, STSMs can be formed. The researchers further demonstrated the speckle-resolved spectral-temporal dynamics of STSMs in real time [79]. For the first time, they captured diverse real-time dynamics of STSMs, including their birth, spatiotemporal interactions, and internal vibrations. Additional studies reveal that nonlinear spatiotemporal coupling, associated with a large average-chirp gradient over the speckled mode profile, plays a crucial role in these dynamics. These findings provide new insights into the complexity of STSMs and create a compelling analogy between STSMs and chemical molecules.

### 3.4 Soliton molecular complexes

A multi-soliton complex is a self-localized state created by the nonlinear superposition of multiple fundamental solitons [209]. In optics, this can appear as a single light beam or pulse formed by the nonlinear combination of solitons moving at the same velocity. This superposition can be either coherent or incoherent, allowing solitons to bind together or remain close due to their identical initial velocities. The dynamical behaviour of soliton complexes represented by SMs in fibre lasers is complex, involving the formation, stabilization, and potential dissociation under specific conditions [9], [210]. Their dynamics are affected by soliton interactions and factors acting in the laser cavity, such as the gain saturation, loss, and dispersion [211]. Soliton “supramolecules” involve the aggregation of multiple soliton molecules to form more complex structures [75].

**Figure 10:**
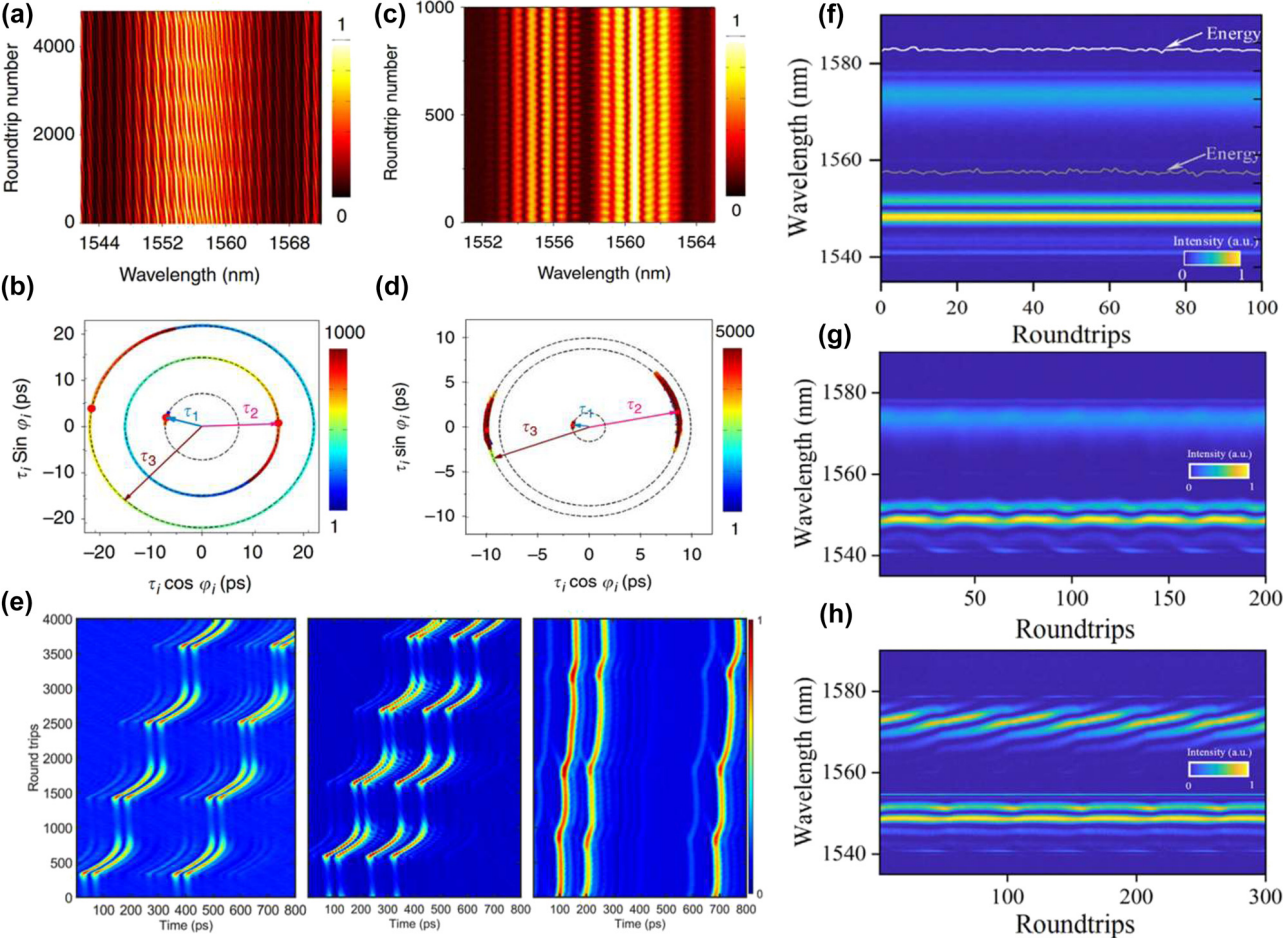
Various dynamics of different types of SM complexes. (a–d) The spectral evolution and interaction plane of sliding-phase and oscillatory-phase 2 + 2 SM complexes, as per Ref. [73] (© Wang et al., Springer Nature 2019); (e) the dynamics of various breather SM complexes, as per Peng et al. [212] (© Wiley-VCH Verlag 2021); (f–h) the spectral evolution of (2 + 1) and (2 + 2) breather dichromatic SM complexes, as shown by Cui et al. [195] (© Wiley-VCH Verlag 2024).

Advancements in real-time spectroscopy techniques have made the above-mentioned DFT technique a powerful tool for observing the transient dynamics of SM complexes. Wang et al. used DFT to show that two soliton pairs can combine to form stable SM complexes of the (2 + 2) type, highlighting differences between intramolecular and intermolecular bonds [73]. Figure 10(a) and (b) illustrate the spectral evolution of the complex of the 2 + 2 type, revealing two distinct spectral fringe patterns and emphasizing the differences in the bond dynamics. From the interaction plane, as displayed in Figure 10(c) and (d), the sliding phase and oscillatory phase can be easily found. Meanwhile, the temporal pulse separation remains nearly constant. The phase changes indicate that the bonds between the soliton pairs are weaker than those within the pairs. In 2021, Peng et al. revealed multibreather SMs and various types of breathing SM complexes, as shown in Figure 10(e) [212]. In (2 + 2) breathing SM complexes, consisting of two bound breather pairs, the intramolecular pulse separations can be either equal or unequal. For the (2 + 1) type, the complex is composed of a breather-pair molecule bound to a single breather. These similar breathing SM complexes can also be intelligently controlled by designing breather-tailored merit functions for use in evolutionary algorithm optimization procedures [101]. As previously mentioned, under the binding mechanism provided by the XPM, dichromatic SMs can be achieved. By further adjusting the pump power, Cui et al. have introduced a new type of dichromatic SM complexes [195], representing a bound state of soliton pulses with two different carrier wavelengths in the fibre laser. Dichromatic SM complexes are sustained by two binding mechanisms: SPM coupling solitons at the same wavelength and XPM coupling them at different wavelengths. The synchronized motion of the 2 + 1 type dichromatic SM complexes results from strong binding between solitons at the same wavelength, as shown in Figure 10(f). Figure 10(g) illustrates the dynamics of the 2 + 1 type vibrating dichromatic SM complexes, while Figure 10(h) depicts the 2 + 2 type vibrating one, showing identical interference fringes at both wavelengths and demonstrating the robustness of the four-soliton bound state.

**Figure 11:**
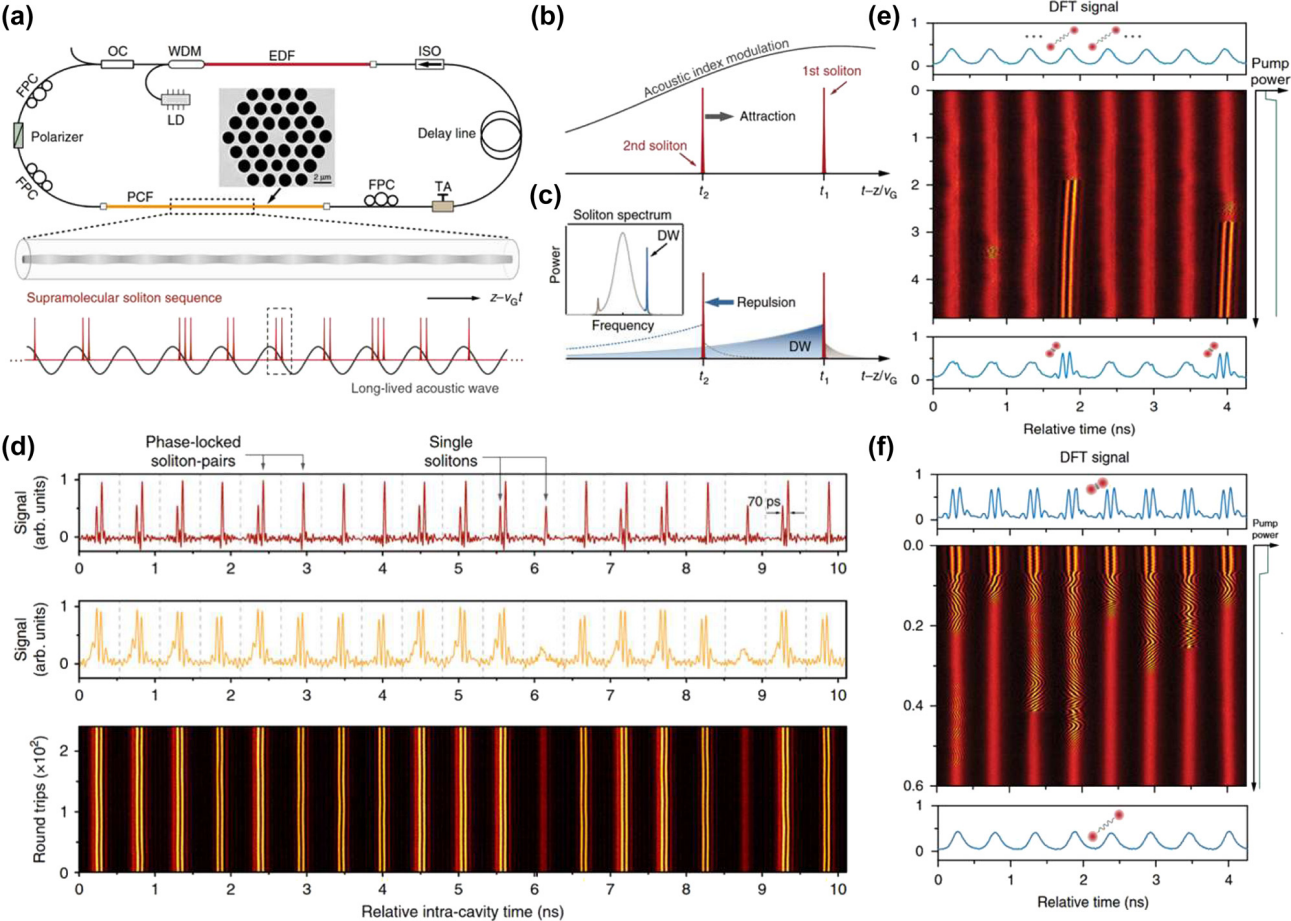
The experimental setup and evolution dynamics of soliton “supramolecules”. (a–d) The experimental setup and evolution dynamics of soliton “supramolecules”, together with the schematic of the optomechanical effect, as per He et al. [75] (© Springer Nature 2019). (e, f) The synthesis and dissociation of SMs in soliton “supramolecules” as per He et al. [34] (© Springer Nature 2021).

Soliton “supramolecules” are complex structures formed through the aggregation of multiple soliton molecules via long-range interactions. Unlike single solitons, the supramolecules exhibit enhanced complexity and diverse dynamical behaviours, with their formation and stability affected by the strength of interactions between SMs, their phase relations, and the nonlinear environment in the laser cavity. In 2019, He et al. demonstrated that long-range forces of diverse physical origins acting between different soliton components can be effectively coordinated in the fibre-laser cavity, resulting in the self-assembly of numerous optical solitons into highly ordered supramolecular structures [75]. The mode-locked fibre laser utilized in their study of soliton supramolecules, together with a schematic of the optomechanical effect is illustrated in Figure 11(a)–(c). A 2-m-long solid-core silicon photonic crystal fibre (PCF) with GHz acoustic core resonance was incorporated into the laser cavity. The long-range binding of multiple solitons within each optomechanical unit results from a balance between attractive and repulsive forces between the solitons. Figure 11(d) presents the temporal traces and DFT signals of soliton supramolecules formed from single solitons and soliton pairs. The study of the soliton supramolecules reveals their potential as unique configurations with long-term stability. The interactions between solitons contribute to their structural flexibility and reversibility, allowing to perform precise optical manipulation of these states. Their stability is primarily governed by long-range interactions that create effective “springs” connecting between the solitons, anchoring them at equilibrium positions.

Conventional mode-locked lasers normally host, at most, only a few solitons, which means that stochastic behaviours involving large numbers of solitons cannot easily be studied under controlled experimental conditions. The mentioned optoacoustic mode-locked fibre lasers proposed by He et al. might be considered as parallel optical-soliton reactors within each of which multiple solitons can be isolated and controlled both globally and individually using all-optical methods [34]. Figure 11(e) illustrates the global synthesis of phase-locked SMs. Following a sudden change in pump power or cavity loss, the long-range binding collapses, causing the two solitons to undergo multiple collisions before forming stable SMs. Figure 11(f) illustrates the dissociation of soliton molecules in parallel reactors. By perturbing the pump power or cavity loss, the molecular bonds collapse, leading to the solitons drifting and diverging randomly before attaining a stable long-range bound state characterized by highly diverse dissociation trajectories. These results suggest possibilities for exploring the dynamics of soliton interactions, as it is now possible to investigate the statistics of multi-soliton systems under controlled conditions. The ability to induce the formation and dissociation of SMs highlights the intricate mechanisms at play in nonlinear optical systems. Ongoing studies of soliton supramolecules naturally focus on understanding their formation and stability. This work may lead to significant advancements in applications, such as optical data storage, ultrafast laser technology, and nonlinear optics in general. As the complexity of these structures is further elucidated, they may reveal new peculiarities of fundamental nonlinear interactions and their practical implications in advanced optical systems.

## 4 Manipulations of soliton molecules

Precise manipulations of SMs suggest novel applications to optical communication [23], [36], fibre sensing [9], precision measurements [213], etc. These capabilities enable more efficient data-transmission schemes and more accurate optical measurements. By externally controlling relevant parameters, such as gain [184], [214], [215], polarization, and dispersion in the laser cavity, the multi-pulse bound states can be precisely tailored on demand, including adjustments of the pulse number, temporal separation, relative phase, spectral shape, mode-locked state, and various transient processes. Currently, the methods for manipulating SMs are becoming increasingly diverse.

### 4.1 Polarization control of soliton molecules

**Figure 12:**
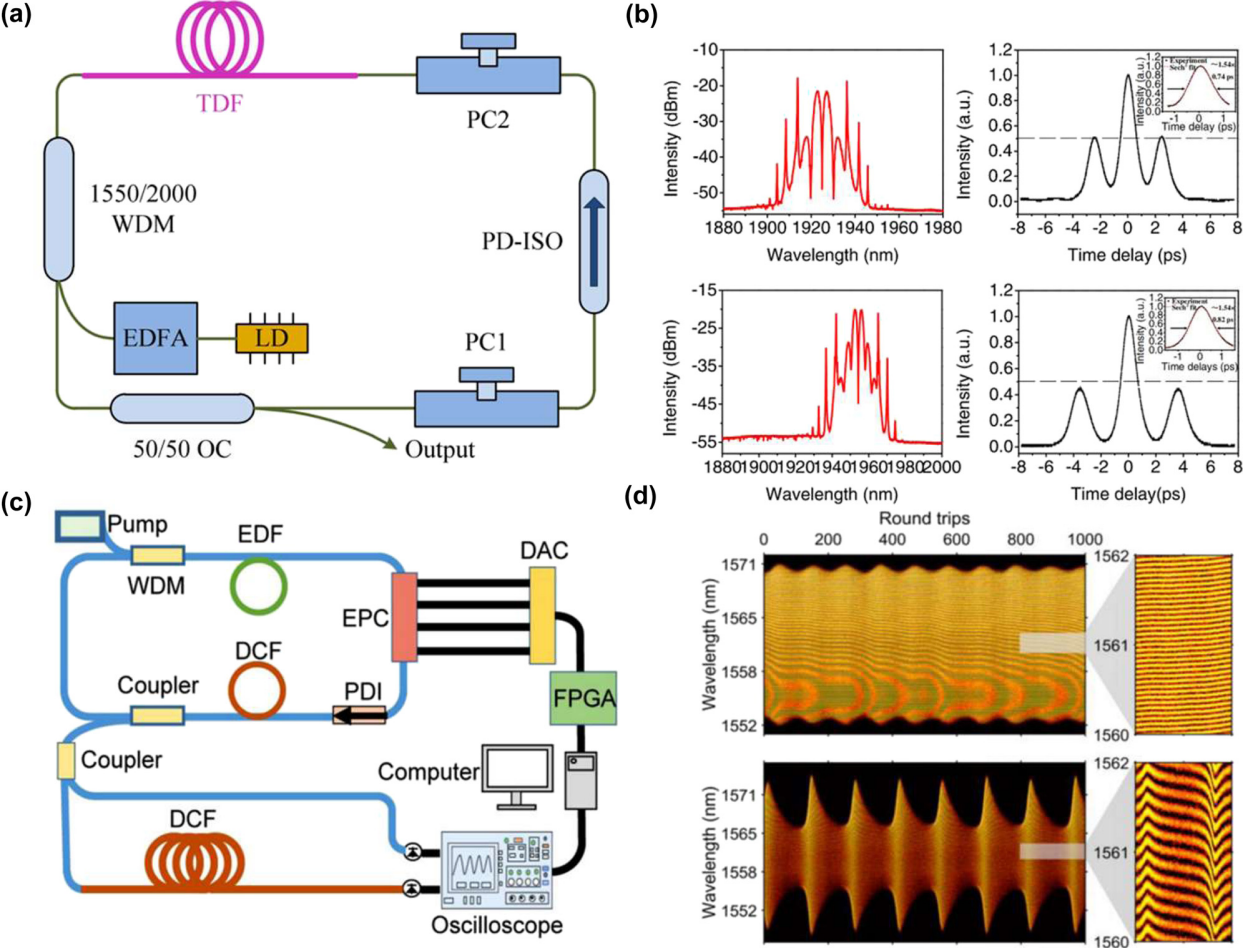
Two managing ways of SMs by polarization control. (a, b) The experimental setup, and the output spectrum of the SMs, realized by adjusting the polarization state (Wang et al. [52] © The Optical Society 2016). (c, d) The experimental setup and spectra for the intelligent generation of breathing SMs in ultrafast fibre lasers (Wu et al. [101] © Wiley-VCH Verlag 2022).

The polarization control of lasers is one of the mainstream methods in current pulse-manipulation techniques. In 2016, Wang et al. adjusted two polarization controllers (PCs) in an all-fibre thulium-holmium co-doped laser running in the NPE mode-locking regime to generate stable SMs, making it possible to adjust the central wavelength of the SMs from 1,920 nm to 1,940 nm, by changing the PC direction [52], as shown in Figure 12(a) and (b). In 2018, Wang et al. achieved the generation of multi-pulse SMs by adjusting the PC and increasing pump power in a passive mode-locked erbium-doped fibre laser based on bismuth-silicate SA [216]. In addition, some intracavity components, such as fibre Bragg gratings and filters [74], can be added to achieve the tuning of the central wavelength. In experiments, electric polarization controllers (EPCs) are typically used to achieve an on-demand design of pulse polarization states. EPCs offer a wide control range, fast response speed, high integration, and ease of operation, making them widely applicable in optical communications, optical experiments, and laser technologies. In recent years, significant progress has also been made in the studies of multi-pulse manipulations through polarization control. In 2015, Andral et al. used evolutionary algorithms to implement the EPC-assisted control of pulse polarization states [217]. Through feedback optimization, they aimed to establish mode-locking in the laser cavity, achieving the intelligent polarization control of the mode-locked state. These studies on polarization control with the help of EPCs suggest new possibilities for the implementation of the polarization control of multi-pulse states. By combining the DFT technique with machine learning algorithms for controlling EPC, breathing SMs can also be intelligently controlled, as shown in Figure 12(c) and (d) [101]. Furthermore, Liu et al. investigated the temporal arrangement and internal dynamics of soliton triplets by means of the EPC-assisted control [115]. They analysed how the polarization adjustment affects the internal dynamic characteristics of the triplets, uncovering the polarization control mechanism for the three-pulse bound states.

### 4.2 Dispersion control of soliton molecules

**Figure 13:**
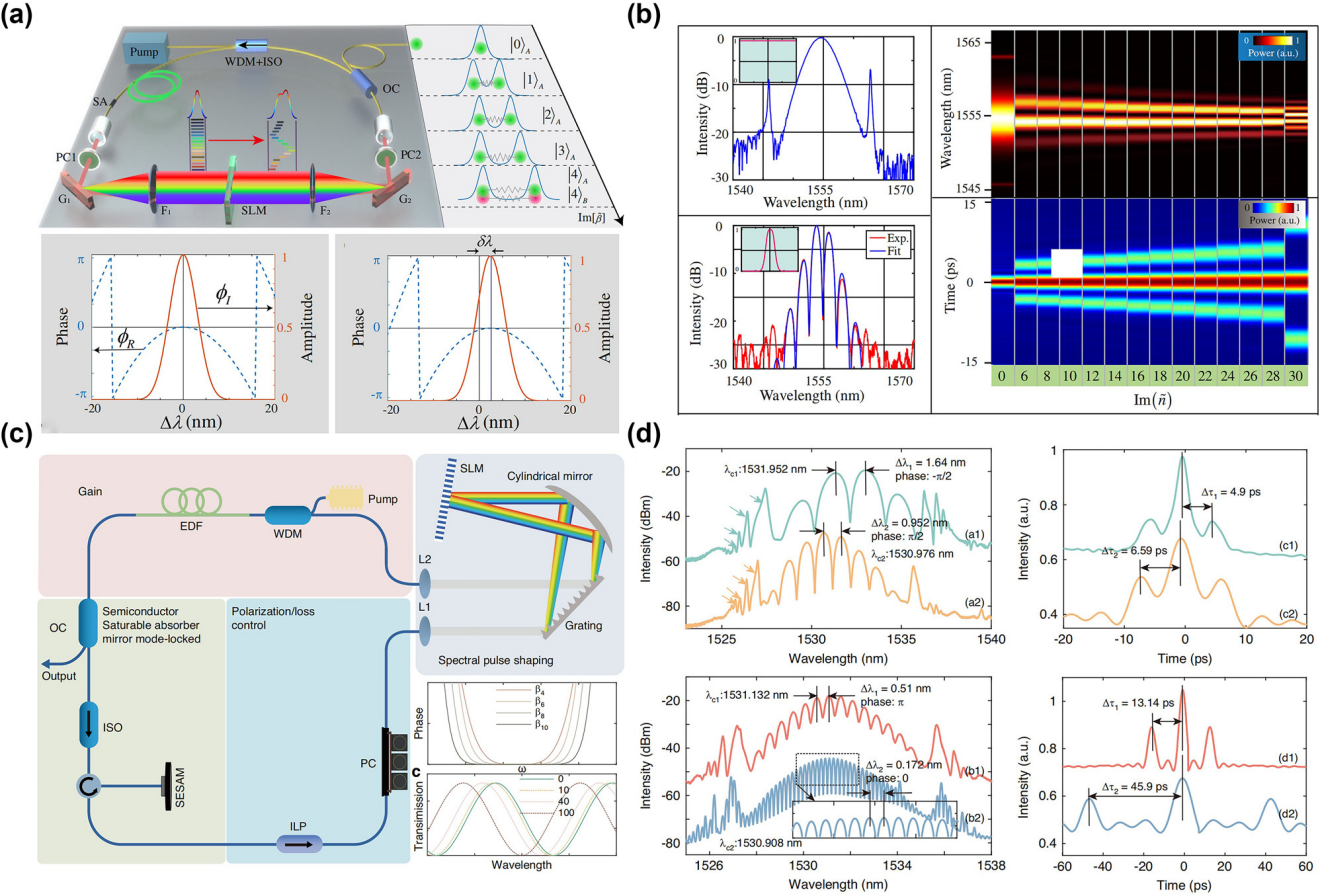
Various dispersion control methods of SMs. (a, b) The experimental setup and results of on-demand generated SMs by adjusting the dispersion loss (Liu et al. [99] © OSA publishing 2022). (c, d) The experimental setup and measured spectra of SMs for adjusting the high even order dispersion (Han et al. [105] © Springer Nature 2024).

The dispersion control of lasers implies adjusting the resonator’s dispersion to modify the pulse output characteristics, offering broad application prospects in optical communications and other fields [218]. Studies have shown that higher-order dispersion has a notable impact on the soliton dynamics in passively mode-locked fibre lasers. Different net higher-order dispersion values in the cavity can affect the formation, shape, spacing, spectral evolution, and stability of SMs [219]. In 2022, Liu et al. used a spatial light modulator (SLM) to control higher-order dispersion, enabling arbitrary switching and encoding of pulse intervals [99]. As shown in Figure 13(a), SLM was placed in the resonator, allowing the customization of the group velocity dispersion and dispersive loss by adjusting parameters of the spectral filtering, thereby achieving the dispersion control of multi-pulse intervals, as displayed in Figure 13(b). Furthermore, Mao et al. have used a programmable pulse shaper (PPS) to control the intracavity group delay to obtain polychromatic SMs [77] and heteronuclear polychromatic SMs compounds [189]. Recently, Han et al. have demonstrated precise control of the generation of solitons and the transition of SMs between different states, such as vibrational, sliding, and mixed ones, by introducing the necessary pure even-higher-order even dispersion by SLM placed inside the cavity, as shown in Figure 13(c) [105]. By adjusting the polarization controller and the value of the intra-cavity eighth-order dispersion, various pure-octic phase-locked SMs were generated, with their measured spectra displayed in Figure 13(d). The spectra exhibit multi-sidebands (indicated by arrows) induced by the large intra-cavity eighth-order dispersion. Furthermore, SMs with different phases were obtained by varying the intra-cavity net eighth-order dispersion. This demonstrates that the phase difference between bound solitons is influenced not only by pump power and cavity length, but also by the intra-cavity high-even-order dispersion [220]. These findings indicate that the intracavity dispersion affects not only the temporal size of SMs, but also the stability and spectral characteristics of the pulses. Thus, by adjusting the dispersion characteristics of the cavity, one can accurately control the SM state. This conclusion is of essential importance for implementing the precise control of the operation of ultrashort pulse lasers.

### 4.3 Gain-modulation control of soliton molecules

Rapidly changing the pump power and adjusting the frequency are effective technical means for the SM control. These methods can essentially affect various SM characteristics, including but not limited to the pulse amplitude, pulse period, pulse frequency [221], [222], as well as the evolution of internal spacing and relative phase between the bound solitons [73].

**Figure 14:**
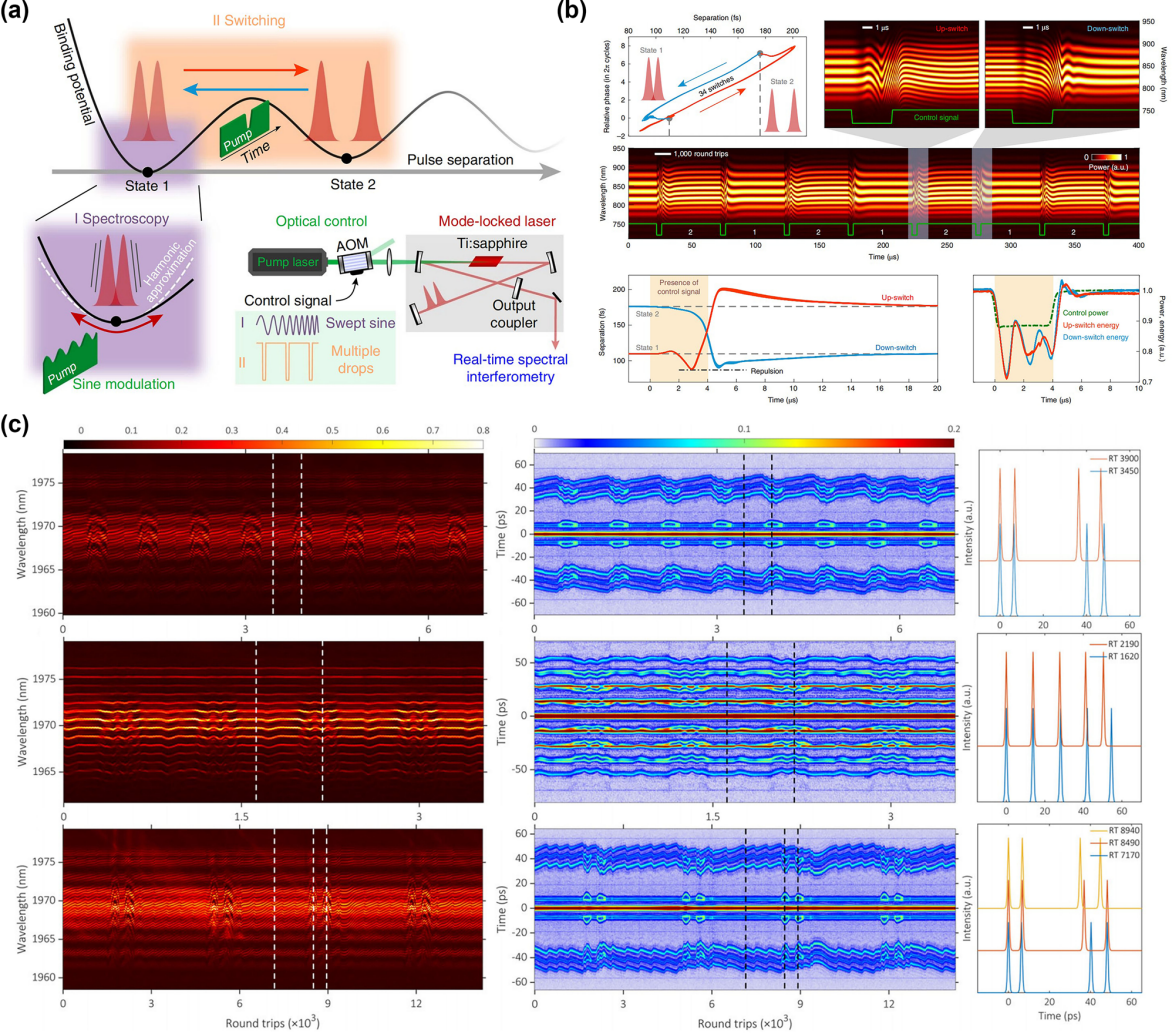
The management of SMs is achieved by quickly changing the pump power and adjusting the frequency. (a, b) Two schemes for optical manipulations of SMs and transitions between different SM states (Kurtz et al. [95] © Springer Nature 2020). (c) The periodic switching of various SM complexes (Zhou et al. [223] © Springer Nature 2022).

In 2019, Kurtz et al. proposed two methods for detecting and controlling the internal dynamics of optical SMs and for switching between different SM states [95], as shown in Figure 14(a) and (b). The first method involves detecting the resonant behaviour and nonlinear characteristics of SMs by applying resonant vibrations through harmonic modulation. By changing the frequency of the pump power modulations, vibrational modes are excited in SMs. The second method is based on the possibility that rapid changes in the pump power can trigger a switch of SM from one stable state into another one. The multi-state switching of SMs is achieved through a non-perturbative approach, leading to attractive applications in ultrafast spectroscopy, information encoding, and logic operations [100]. The results show that, under specific modulation conditions of the pump intensity, the pulsation period of the SM spectrum can be precisely adjusted by the pump-modulation frequency. The amplitude of the SM pulsations and the evolution of the relative phase between the solitons are strongly affected by the pump modulation frequency. For soliton triplets, gain and polarization control can be implemented to artificially manipulate the internal dynamics of this tri-pulse structure, resulting in a diversity of intra-molecular evolutions [115]. By rapidly changing the pump power and adjusting the frequency, periodic switching of SM complexes has also been observed, as shown in Figure 14(c) [223]. In addition, collisions of drifting solitons or the alteration of saturable absorption parameters can also trigger single and multiple switching of SM complexes. These findings provide essential guidance for a deeper understanding of the generation, stability enhancement, and all-optical manipulations of SMs, as well as their applications in areas such as data coding [99], [224].

### 4.4 Soliton molecules control by external light injection

**Figure 15:**
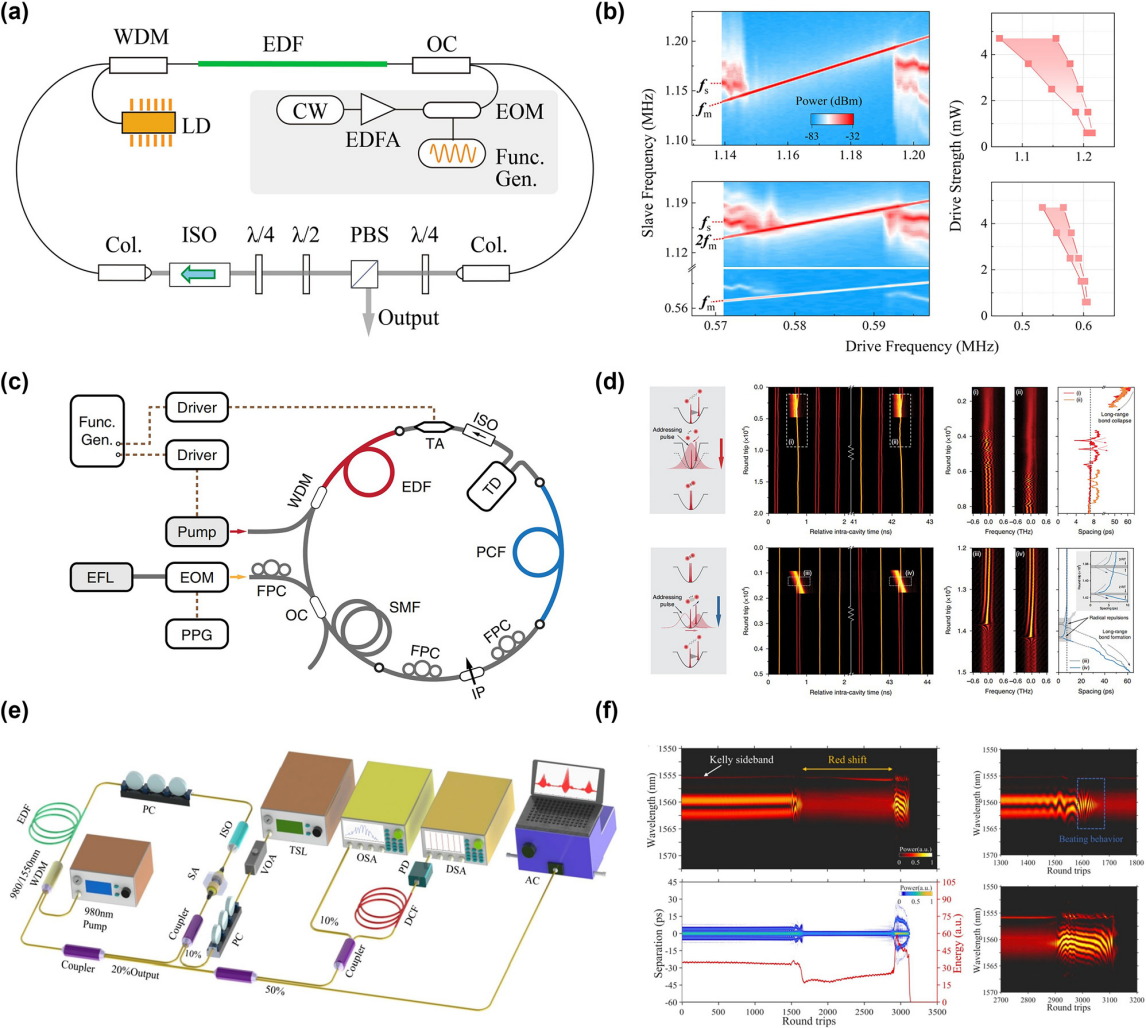
The control of SM by the external light injection. (a, b) Synchronization of internal vibrations in SMs through injection of optically modulated CW. Direct observation of the synchronization process using the BOC technique (Zou et al. [96] © OSA Publishing 2022). (c, d) Synthesis and dissociation dynamics of SMs with the help of addressing pulses (He et al. [34] © Springer Nature 2021). (e, f) Spontaneous collapse and rebuilding-up of SMs triggered by injected CWs (Chang et al. [225] © Elsevier Ltd 2022).

The control of SMs by external optical injection is an advanced technical method that utilizes the synchronization of optical solitons in laser oscillators [95]. As illustrated in Figure 15(a) and (b), Hu et al. applied sinusoidal modulation to a continuous optical signal, using an electro-optic modulator, injecting the signal into a passively mode-locked erbium-doped femtosecond fibre laser. By using the BOC technology for achieving sub-femtosecond resolution in real-time monitoring methods, they demonstrated directional traction and locking of the vibration frequency in the dynamical SM states [96]. Thus, they observed and controlled subtle interaction modes inside SMs, further achieving synchronized control of the SM internal dynamics. Continuous-wave (CW) lasers generated by electro-optic modulators can be utilized for external optical injection. Accordingly, He et al. employed CW lasers integrated with electro-optic modulators, synchronizing them to the laser-cavity’s temporal lattice, using a programmable pattern generator [34], as shown in Figure 15(c) and (d). By precisely timing the address pulses to interact with the solitons in the target time slots, it was possible to reversibly and selectively edit each soliton element without disturbing other solitons, thus developing a method of controllable manipulations of the SM internal dynamics. As shown in Figure 15(e) and (f), Chang et al. used the CW optical injection to perturb the gain dynamics of the SMs in the fibre-cavity laser, and observed spontaneous collapse and revival of the SMs [225]. During the collapse process, the double solitons either disappeared directly or decayed into an intermediate single-soliton state. In the revival phase, complex soliton interactions and oscillations remerged. This method of controlling SMs by external optical injection not only enhances the understanding of SM generation and stability, but also provides valuable insights into the field of all-optical manipulations and the potential application of SMs.

### 4.5 Data encoding by means of soliton molecules

**Figure 16:**
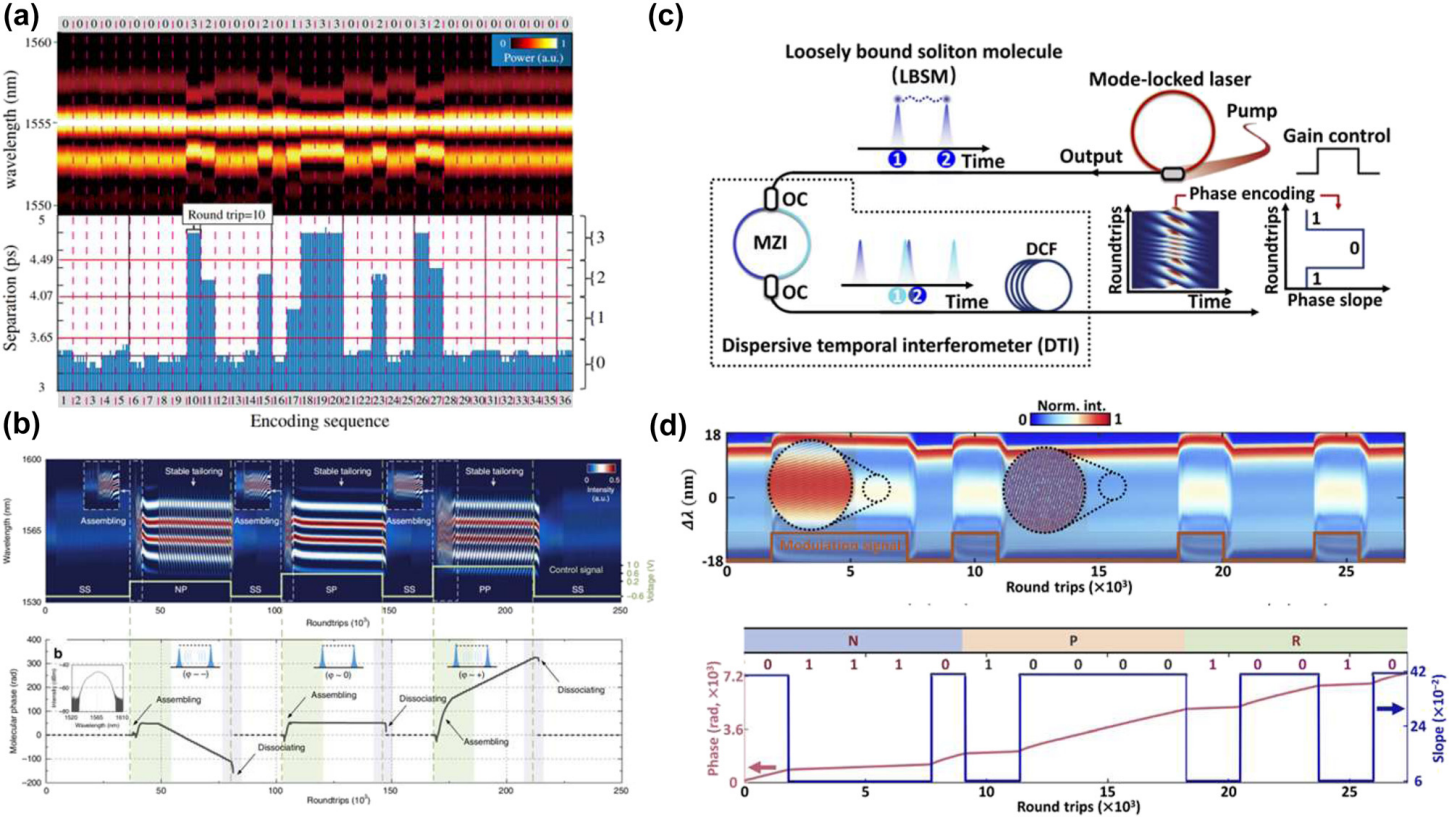
Various ways of data encoding based on SMs. (a) The coding realized in terms of different SM temporal separations (Liu et al. [99] © OSA Publishing 2022). (b) The programmable phase modulation based on the phase-customized quaternary of internally assembled dissipative SMs (Liu et al. [100] © Springer Nature 2023). (c, d) The programmable phase-coded modulation of loosely bound SMs by means of the gain control (Yang et al. [224] © AIP Publishing LLC 2024).

The variation in SM phase difference and temporal spacing can be used to encode data [79], which imparts to them a potential application value in the fields of optical communications and data processing. In nonlinear optics, SMs are structures composed of multiple solitons that can self-assemble like particles and form stable bound states under the action of balanced interaction forces [99]. Due to the strong covalent bond results in internal phase correlation, the time separation of tightly bound SMs can be easily controlled and used for data encoding. In 2023, Liu et al. developed a temporal separation-based quaternary encoding format that relies on the controllable internal assembly of dissipative SMs [99], as illustrated in Figure 16(a). By controlling second-order group-velocity dispersion and dispersion losses, it is possible to select desired values of temporal separation in SMs. Lately, Liu et al. modulated the gain of the laser using an arbitrary function generator to precisely control the four phase evolution states of the SMs, thereby defining a highly distinguishable quaternary encoding format [100]. By designing specific telecom signals to adjust the pump pulse power, they controlled the relative phase changes of the dual-pulse bound state, achieving phase-customized assembly and encoding of multi-pulse bound states, as shown in Figure 16(b). This study demonstrates the feasibility and high fidelity of gain-controlled pulse phase variations and validates the effectiveness of phase-customized quaternary encoding, as well as its potential for development in the field of all-optical storage. The weak phase correlation of loosely bound SMs complicates the fringe resolution of spectrum evolution with standard measurement technologies [226], hindering the analysis of the intricate dynamics of loosely bound SMs. Yang et al. employed a dispersive temporal interferometer, as shown in Figure 16(c), to elucidate the dynamics of loosely bound SMs in mode-locked fibre lasers and to uncover the associated phase evolution mechanism [224]. The dispersive temporal interferometer produces real-time fringe-resolved spectra of the phase evolution of SMs by generating a duplicate of SMs and reducing the temporal gap between the individual solitons of loosely bound SMs. They perform programmable phase-coded modulation of loosely bound SMs through gain control as displayed in Figure 16(d), based on an understanding of its phase dynamics. These findings demonstrate the potential of SMs as data carriers in optical systems, particularly for applications to optical data processing, optical communications, and all-optical storage [73], [227].

## 5 Conclusion and outlook

In this review, we summarize the latest experimental results for SMs (soliton molecules) in mode-locked fibre lasers. As promising bound states of multiple pulses, SMs are of potential significance for use in high-capacity telecommunications. These stationary pulse pairs arise from balanced forces in fibre lasers, forming bound states with fixed temporal separations and particular values of the relative phase. SMs are generic states in fibre lasers, independent of specific cavity details, such as the operation wavelength, intracavity dispersion regime, or mode-locking technique. However, their temporal pulse separation is highly sensitive to the cavity parameters (in particular, the saturation gain). SMs readily self-trap from two-soliton inputs with the temporal distance between the solitons, which can exceed the SM’s proper temporal size by up to two orders of magnitude, depending on the strength of the dominant effects. In addition to the relatively simple basic temporal SMs, the complex dissipative dynamics in fibre lasers can lead to a variety of other multi-soliton states and phenomena, including polychromatic and spatiotemporal SMs, as well as SM complexes. These SMs demonstrate diverse scenarios of the nonlinear behaviour, including the build-up, pulsations and interactions. They are not only objects of fundamental interest but also offer a considerable potential for applications related to chaos, optical storage, and optical switching.

The versatility of fibre laser cavities enhanced the current understanding of multi-pulse interaction mechanisms and provides valuable insights into the generation and control of multiple solitons through diverse approaches, such as gain modulation, polarization control, dispersion management, and photomechanical effects. However, the large number of essential experimental parameters and the limited set of practically available characterization tools hinder the progress of studies in this field, making a complete analysis of the soliton dynamics a challenging objective. In particular, while addressing pulse trains and individual pulses, it is difficult to achieve a precise description of soliton complexes. Looking forward, more experimental observations and theoretical analyses may be expected, following the progress in the development of optical measurement methods, including direct access to the phase information. Recent breakthroughs in UTO techniques enable the real-time observation of the build-up and evolution of SMs, which may be applied and tested in more systems and situations. Moreover, trials on long-distance transmission of SMs and encoding multiple solitons – in particular, by means of polarization-state manipulations – may pave the way towards real-world applications.

SMs may find important applications in optical communication [35], [36], [37], [38]. Their stability and resistance to interference can significantly improve the capacity and reliability of communication systems. Furthermore, SMs with multidimensional parameter space can be used in high-sensitivity fibre-optic sensors to detect subtle environmental changes, such as temperature, pressure, and stress variations [228]. They can also be utilized in high-resolution biological imaging techniques [229], providing detailed structural and functional information about biological tissues. SMs with multidimensional parameter space may also serve as data carriers in optical computing [230], enabling high-speed parallel algorithms. In principle, the quantum properties of SMs may be used for quantum data transmission and quantum computing, enhancing processing speeds and data-handling capacity [17].

In spite of the significant progress achieved in the SM studies, there are conspicuous challenges. Achieving long-term stability and on-demand SM is still difficult [231], [232], particularly in practical applications. Precisely controlling SMs in complex environments, especially in the presence of strong noise and disturbances, requires more robust control systems. This, improvements of multidimensional control of SMs – including the synchronization in time, space, and frequency – remain a key direction for the ongoing research.

The integration of artificial intelligence (AI) in controlling SMs in fibre lasers represents a cutting-edge research area [233], [234]. AI can analyse operational data from fibre lasers and build efficient models to predict the SMs’ behaviour [235]. For example, machine learning algorithms (such as physics-informed neural networks [236], [237]) can extract patterns and rules from experimental data, optimizing the design and parameter settings of fibre lasers. AI can be used to optimize the working parameters of the lasers, such as pump power, fibre length, and nonlinear effects. Through optimization algorithms, such as genetic ones [238], [239] or particle swarm optimization [240], the best parameter combinations may be found to achieve precise control over SMs. AI’s real-time data analysis capabilities can monitor soliton dynamics in fibre lasers. By means of real-time analysing, AI can adjust the laser’s operating conditions to ensure the SMs stability and desired characteristics [94]. An AI-based adaptive control system can be designed to continuously learn and adjust in response to environmental and system changes in fibre lasers, thus maintaining the stability and proper performance of SMs. AI can also be employed for computer simulations aimed to predict SM behaviour under varying conditions [241]. The simulations can help to optimize the design of fibre lasers, improving their performance and stability. In multiscale modelling of fibre lasers, AI can assist in integrating models across different scales (e.g., the microscopic soliton behaviour and macroscopic laser performance), to enable a more comprehensive control of the system.
